# δ-Tocotrienol preconditioning improves the capability of bone marrow-derived mesenchymal stem cells in promoting wound healing by inhibiting BACH1-related ferroptosis

**DOI:** 10.1038/s41420-023-01653-1

**Published:** 2023-09-22

**Authors:** Xiao He, Dawei Wang, Yi Yi, Yufang Tan, Min Wu, Haiping Wang, Weijie Hu, Hongbo Chen, Qi Zhang, Yiping Wu

**Affiliations:** Department of Plastic Surgery, Tongji Hospital, Tongji Medical College, Huazhong University of Science and Technology, Wuhan, 430030 Hubei China

**Keywords:** Mesenchymal stem cells, Cell death

## Abstract

Wound healing is a complex physiological process for maintaining skin integrity after a wound. Bone marrow-derived mesenchymal stem cells (BMSCs) are excellent cellular candidates for wound healing, which could be enhanced by exogenous stimulation. We aimed to explore the role of δ-Tocotrienol (δ-TT) in BMSC ability of wound healing. Firstly, transcriptome and single-cell analysis were used to explore the genes and pathways related to ferroptosis in wound tissues. In vitro, cell proliferation, migration, and angiogenesis of δ-TT-BMSCs were detected. In addition, qRT-PCR and immunofluorescence (IF) were applied for observing the promoting wound healing ability of δ-TT-BMSC conditioned medium (CM) on NIH-3T3 and PAM-212 cells. The level of ferroptosis was determined by the mitochondrial membrane potential and total/lipid reactive oxygen species (ROS) in the cells and the morphological changes of mitochondria were observed by transmission electron microscope. The BTB and CNC homology 1 (BACH1) expression and activation of the PI3K/AKT signaling pathway were detected by IF and western blot (WB). The effect of δ-TT-BMSCs on wound healing was observed in vivo. The regulatory mechanism of δ-TT-BMSCs on ferroptosis was verified by IHC and IF staining. In vitro, δ-TT-BMSCs declined the level of lipid ROS in NIH-3T3 and PAM-212 cells and enhanced mitochondrial membrane potential. In vivo, δ-TT-BMSCs promoted wound healing in mice by decreasing ferroptosis. In terms of mechanism, δ-TT-BMSCs inhibited the expression of BACH1 and activated PI3K/AKT signaling pathway. This study demonstrated the ability of δ-TT-BMSCs to promote wound healing by inhibiting BACH1-related ferroptosis. In addition, PI3K/AKT signaling pathway was activated by δ-TT-BMSCs and could be involved in wound healing. δ-TT-BMSCs might be a promising strategy for treating wounds.

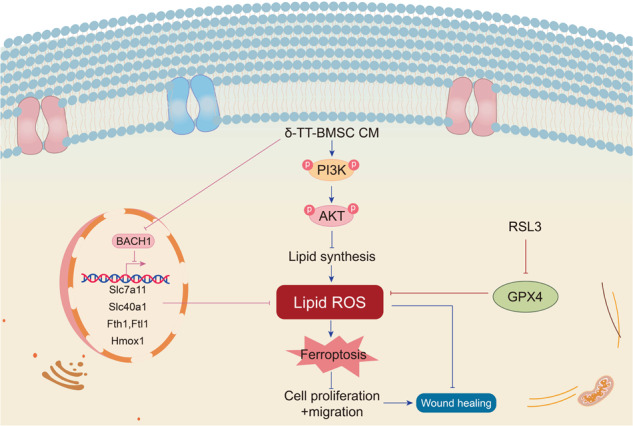

## Introduction

Wound healing is a complex process that occurs in four distinct stages: the hemostatic phase, the inflammatory phase, the proliferative phase, and the remodeling phase [1]. Wounds contain a diverse array of cell types, including fibroblasts, keratinocytes, and endothelial cells. The transforming growth factor-β (TGF-β) family, the platelet-derived growth factor (PDGF) family and angiogenic factors play a role in wound healing [2–4]. Chronic ulcers or excessive scarring can develop due to the loss of control at any stage of the wound-healing process or as a result of personal factors such as advanced age or uncontrolled diabetes [3]. Unfortunately, these poor prognoses continue to pose numerous challenges, representing a significant area of unmet clinical need. Bone marrow-derived mesenchymal stem cells (BMSCs) have enormous potential in tissue healing and regenerative medicine, due to their capacity for self-renewal, cell type differentiation, and secretion of a variety of bioactive substances, bone marrow-derived mesenchymal [4]. BMSCs have positive paracrine effects on cells involved in wound healing (keratinocytes, fibroblasts, and vascular endothelial cells) and thus promote wound healing [5]. The limited migration and secretion capacity of BMSCs, along with their low survival rate, hinder their widespread application. Gene modification and preconditioning interventions can improve wound healing promoting effects of BMSCs. For instance, Yu et al. discovered that BMSC exosomes preconditioned with atorvastatin accelerated the healing of diabetic wounds by up-regulating miR-221-3p and improving endothelial cell biology via activating AKT/eNOS pathway [6]. Similarly, Li et al. found that TGF-β3 gene-modified BMSCs not only promoted wound healing but also reduced scar tissue formation in a rabbit model [7].

Ferroptosis is an iron-dependent cell death, characterized by cytological changes, such as a decrease or loss of mitochondrial cristae, a rupture of the outer mitochondrial membrane, and a loss of mitochondrial membrane cohesion [8, 9]. The loss of the plasma membrane’s selective permeability is the caused by the onset of intense membrane lipid peroxidation and oxidative stress [10]. While the majority of recent research has regarded ferroptosis as a cancer treatment, the effect of ferroptosis on wound healing is still unclear. Ferroptosis can occur in response to injury, irritation, or the presence of individual-specific factors like diabetes, and can significantly affect the healing process [11]. Multiple factors and pathways regulate wound ferroptosis. Ferrostatin-1 (Fer-1), ferroptosis inhibitor, accelerated wound healing by activating the anti-inflammatory PI3K/AKT signaling pathway. Li et al. identified changes associated with ferroptosis in a diabetic rat model [12]. By regulating lipid metabolism and cellular differentiation, BACH1 has been shown to control ferroptosis, as demonstrated by research by Namgaladze et al., who found that silencing BACH1 decreased lipid peroxidation and increased macrophage resistance to ferroptosis [13, 14]. Macrophages are crucial to the healing process at every stage, and their dysfunction impair wound healing. However, more research is needed to understand the role of BACH1 in wound ferroptosis.

Tocopherols (TPs) and tocotrienols (TTs) are the two groups of substances that make up natural vitamin E. In comparison to TPs, TTs exhibit greater antioxidant, anticancer, and hypocholesterolemic properties [15]. δ-TT is the most effective TT at reducing inflammatory mediators, followed by γ- and α-TT [13]. δ-TT effectively reduces inflammatory mediators through the induction of apoptosis and paraptosis in prostate cancer cells via the endoplasmic reticulum (ER) stress and autophagy pathways [14]. δ-TT has been found to trigger reactive oxygen species (ROS) production in cancer cells, leading to its anticancer effects. Raimondi et al. investigated that δ-TT induced epithelial parapoptosis in human melanoma cells. δ-TT could cause endoplasmic reticulum expansion and mitochondrial swelling, and this mitochondrial dysfunction led to excessive reactive oxygen species (ROS) production [16]. However, δ-TT shielded osteoblast MC3T3-E1 and osteoblast MLO-Y4 cells from tert-butyl hydroperoxide (t-BHP)-induced oxidative damage by reducing intracellular ROS levels and increasing the glutathione (GSH)/oxidized glutathione (GSSG) ratio, with a connection to the PI3K/AKT-Nrf2 signaling pathway [17]. This suggested that different cells responded differently to the induction of ROS by δ-TT. Wound healing could also benefit from the use of δ-TT. In a mouse model with a splint wound, Hoff et al. discovered that the controlled release of δ-TT derivative lithotriene from bacterial nanocellulose accelerated wound healing and improved the quality of newly formed tissue [18]. It was interesting to note that Casati et al. discovered that δ-TT could improve BMSC recruitment and promote MC3T3-E1 differentiation and migration [19]. However, there is limited research on the role of δ-TT in wound healing. Our study investigates for the first time the specific mechanism by which δ-TT preconditioning BMSCs to accelerate wound healing. We revealed that δ-TT inhibits ferroptosis, possibly through down-regulation of BACH1 expression and activation of the PI3K/AKT signaling pathway. Overall, δ-TT preconditioning BMSCs may be a novel method to improve wound healing.

## Results

### Identification of the genes and pathways related to wound ferroptosis

**Fig. 1:**
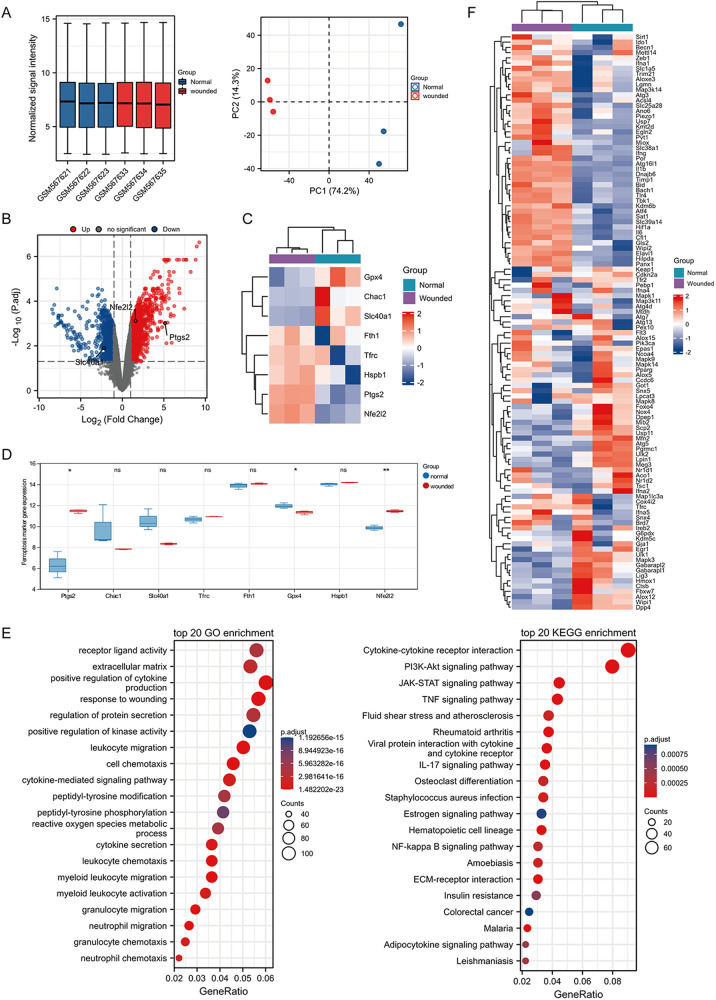
Identifying the genes and pathways related to wound ferroptosis via the transcriptome data. **A** The boxplot of the normalized data and PCA results for two samples. **B** The volcano plot was constructed using the fold change values and P-adjust. Red dots indicated up-regulated genes and blue dots indicated down-regulated genes. **C** Heat map of ferroptosis-related gene expression, in which different colors represented the expression trend in different samples. **D** The analysis result of ferroptosis-related genes. **E** Enrichment analysis of differentially expressed gene. **F** Heatmap of ferroptosis driver gene in two groups. *p < 0.05, **p < 0.01.

**Fig. 2:**
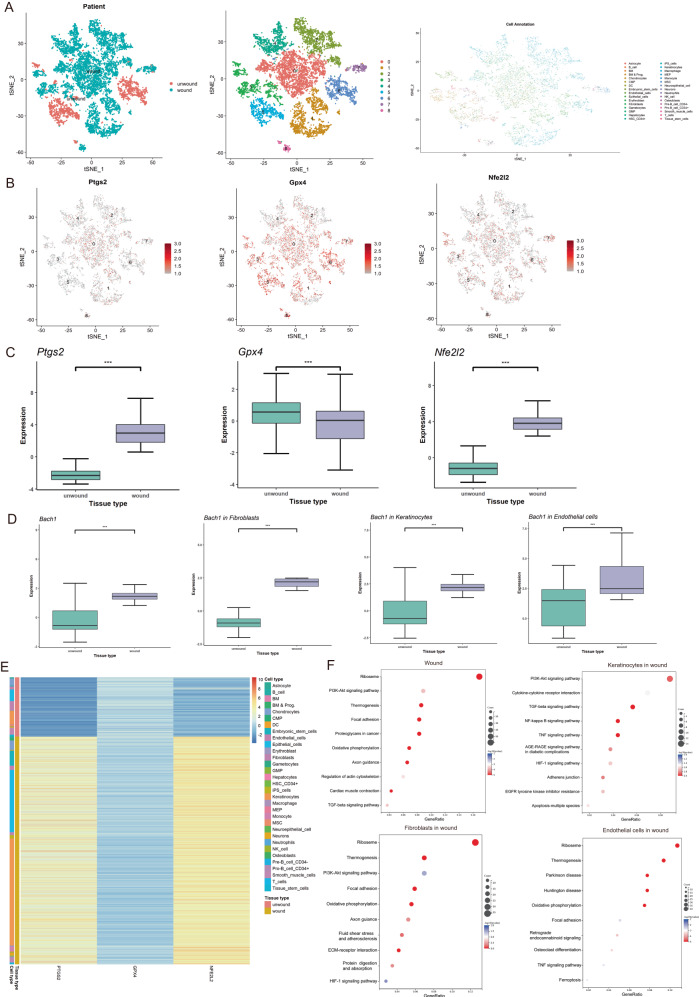
Identifying the genes and pathways related to wound ferroptosis via single-cell sequencing data. **A** The t-SNE scatter diagram of single-cell transcriptome expression. **B** Candidate gene expression patterns at the single cell level. Each point represented a cell, and the deeper the color, the higher the expression. Gray denoted no expression and red indicated expression. **C** The expression difference of candidate genes at the single cell level. **D** The expression difference of BACH1 at the single cell level. **E** The heat map of candidate genes and phenotypes. **F** Enrichment analysis of differentially expressed gene. ***p < 0.001.

We downloaded microarray data from the GEO database for different analyses (Fig. 1A). GPX4 expression was down-regulated and PTGS2 and NFE2L2 expression were up-regulated in the wound group compared to the normal group, indicating ferroptosis phenomenon in the wound group (Fig. 1B–D). The KEGG analysis showed that the differentially expressed genes were mainly associated with the PI3K/AKT signaling pathway (Fig. 1E). A heat map of ferroptosis driver genes in two groups was shown in Fig. 1F. The expression of BACH1 was found to be higher in the wound group. we further obtained a single-cell sequencing dataset GSE153596 from the GEO database. The raw data from GSE153596 were converted into an expression matrix, cells were clustered using the t-SNE algorithm for dimensionality reduction, and the pre-processed single-cell data were annotated with cell types using the SingleR package (Fig. 2A). At the single cell level, GPX4 expression was down-regulated while PTGS2 and NFE2L2 expression was up-regulated in the wound group (Fig. 2B, C). Ferroptosis was found to occur more frequently in wound tissue than in normal tissue and was predominantly present in keratinocytes, fibroblasts, and vascular endothelial cells. BACH1 was highly expressed in wound tissues and keratinocytes, fibroblasts, and vascular endothelial cells with wound site, compared to normal tissues (Fig. 2D). A heat map of ferroptosis marker genes in association with phenotype was shown in Fig. 2E. Based on KEGG analysis, these differentially expressed genes were mainly related to PI3K/AKT signaling pathway in wound tissues, especially in keratinocytes and fibroblasts (Fig. 2F). Thus, ferroptosis is present more frequently in wound tissue and is associated with BACH1 and PI3K/AKT pathways, and with a higher incidence of ferroptosis in keratinocytes and fibroblasts.

### Identification and culture of BMSCs

**Fig. 3:**
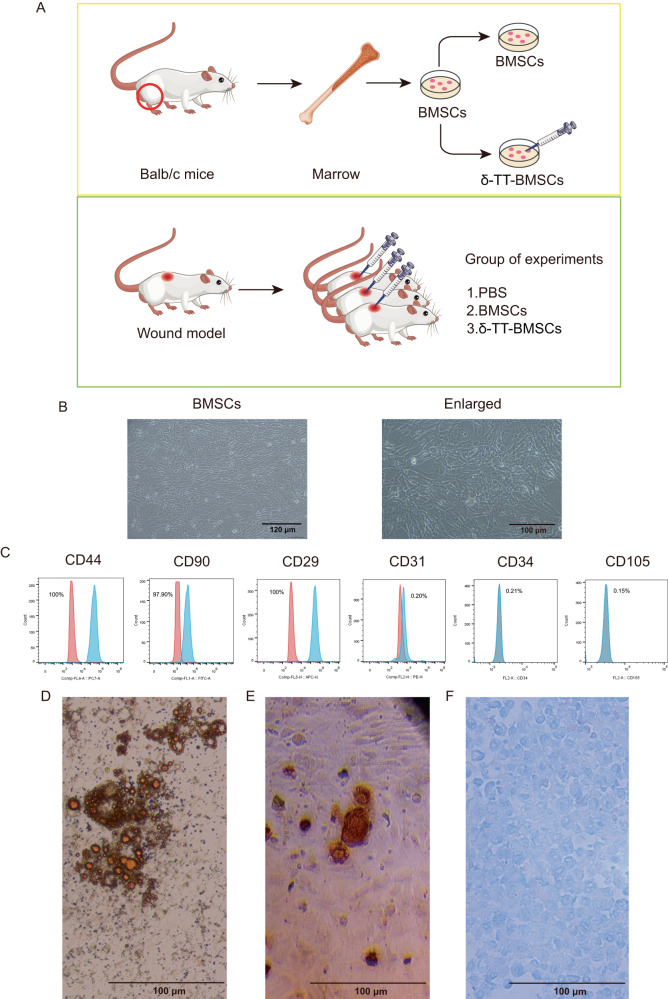
Identification of BMSCs. **A** The flow diagram of the experiment. Wound mice were randomly divided into 3 groups that were treated with PBS, BMSCs, and δ-TT-BMSCs, respectively. **B** Morphology of BMSCs at passage 3 (magnification: ×100, Bar = 120 μm). **C** FCM analysis of the expression of BMSC surface markers, which were positive for CD44, CD90, and CD29 and negative for CD31, CD34, and CD105. **D** Oil-red O staining of adipogenesis differentiation of BMSCs (magnification: ×200, Bar = 100 μm). **E** Alizarin red staining of osteogenic differentiation of BMSCs (magnification: ×200, Bar = 100 μm). **F** Alcian blue staining of chondrogenic differentiation of BMSCs (magnification: ×200, Bar = 100 μm).

Next, the in vitro and in vivo assays were conducted (Fig. 3A). The isolated BMSCs possessed a spindle-shaped and fibroblast-like morphology (Fig. 3B). FCM results confirmed that the obtained cells were positive for mesenchymal stem cell markers CD44, CD90, and CD29, and were negative for endothelial cell markers CD31, CD105, and hematopoietic lineage marker CD34 (Fig. 3C). The results of Oil-Red O staining revealed that differentiating cells adopted an adipocyte-like morphology and contained sizable lipid droplets within the cytoplasm (Fig. 3D). The osteogenic differentiation assay demonstrated that some BMSCs formed colonies-like clusters and grew with red-layered mineralized nodules appeared (Fig. 3E). Finally, the results of alcian blue staining indicated positive results for chondrogenic differentiation as it showed the accumulation of proteoglycans in cartilage (Fig. 3F). Thus, the morphology and biomarker analysis both conclusively demonstrated that the isolated cells were indeed BMSCs.

### δ-TT treatment increased BMSC proliferation and migration

**Fig. 4:**
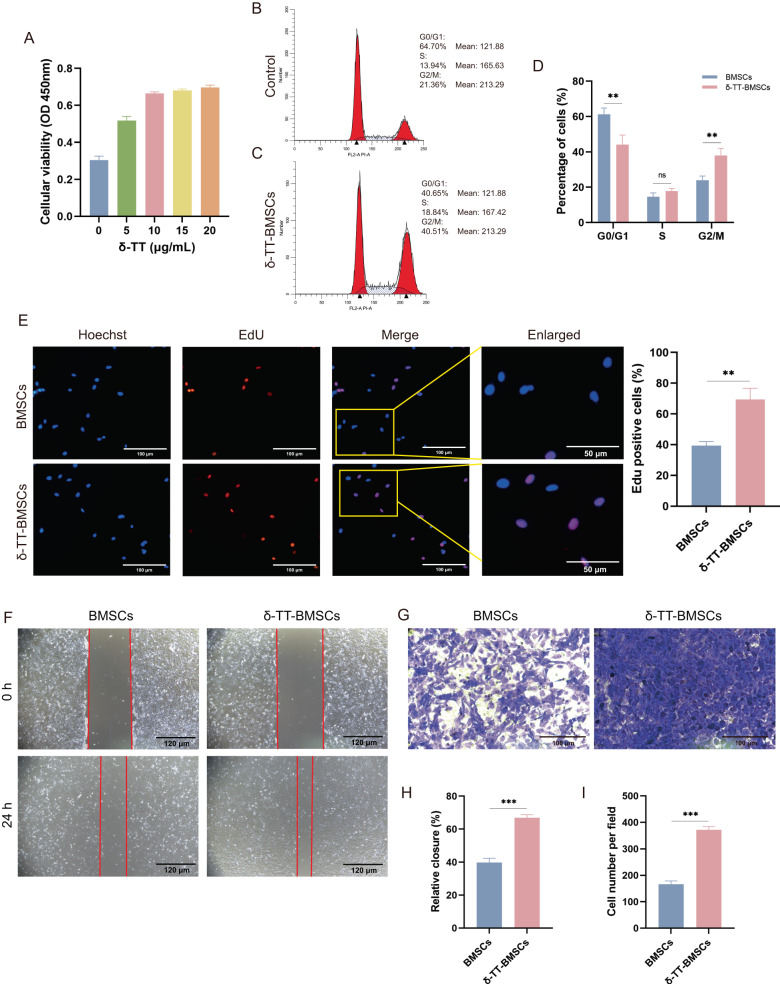
δ-TT promoted the proliferation and migration of BMSCs. **A** Screening the optimum amount of δ-TT to precondition the BMSCs. **B**, **C** Representative illustrations of the cell cycle obtained from flow cytometry analysis. **D** The distribution of cell cycles in BMSCs and δ-TT-BMSCs. **E** EDU staining was utilized to detect cell proliferation in BMSCs and δ-TT-BMSCs (magnification: ×200, Bar = 100 μm). **F** Representative illustrations of BMSC metastasis in the scratch assay (magnification: ×100, Bar = 120 μm). **G** Representative illustrations of BMSC metastasis stained with crystal violet (magnification: ×200, Bar = 100 μm). **H** Quantitative analysis of the percentage of scratch area in scratch wound healing assay. **I** Quantitative analysis of migrated cells in a transwell assay. **p < 0.01, ***p < 0.001.

The proliferation of BMSC was assessed using the CCK8 after 24 h preconditioning with different δ-TT concentrations. BMSC proliferation peaked at a δ-TT concentration of 10 μg/mL and did not increase significantly beyond this concentration (Fig. 4A). Therefore, we chose 10 μg/mL δ-TT to precondition BMSCs. The FCM analysis revealed that the distribution of cells in the G1 phase was significantly decreased after δ-TT treatment compared to the control group, while the distribution of cells in the G2 phase was significantly increased (Fig. 4B–D). In addition, the increase in the number of BMSCs with positive EDU staining in the δ-TT-BMSC group suggested that δ-TT might promote BMSC proliferation (Fig. 4E). In the scratched assay, the δ-TT-BMSCs group had a stronger migration ability compared to the control group (Fig. 4F, H). The transwell assay also revealed that there were significantly more migrated cells in the δ-TT-BMSC group compared to the control group (Fig. 4G, I). These findings suggested that δ-TT could promote BMSC migration.

### δ-TT-BMSCs promoted angiogenesis

**Fig. 5:**
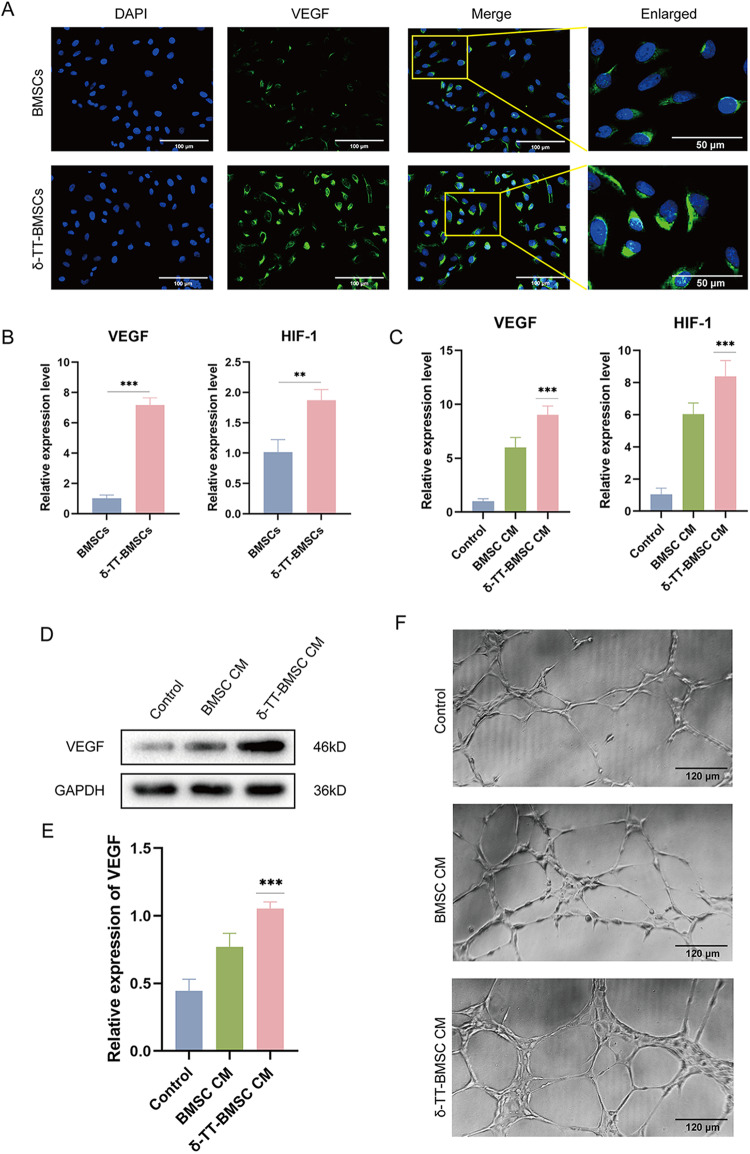
δ-TT-BMSCs promoted angiogenesis. **A** IF was utilized to show the VEGF expression in BMSCs and δ-TT-BMSCs (magnification: ×200, Bar = 100 μm). **B** qRT-PCR results showed the expression of VEGF and HIF-1 in BMSCs and δ-TT-BMSCs. **C** qRT-PCR results showed the expression of VEGF and HIF-1 in C166 cells. **D**, **E** The western blot results of VEGF protein in C166 cells and related quantitative analysis. **F** Tube formation of C166 cells in the control, BMSC CM, and δ-TT-BMSC CM groups (magnification: ×100, Bar = 120 μm). *p < 0.05, **p < 0.01, ***p < 0.001.

The angiogenesis effect of BMSCs was critical for wound healing. Firstly, IF results revealed that the δ-TT-BMSCs group showed significantly higher levels of positive VEGF expression than the control group (Fig. 5A). The qRT-PCR results showed that δ-TT significantly increased the mRNA expression levels of HIF-1 and VEGF in BMSCs (Fig. 5B). Meanwhile, HIF-1 and VEGF mRNA in the BMSC CM group were higher than those in the control group and were highest in the δ-TT-BMSC CM group (Fig. 5C). WB assay also showed a consistent trend of HIF-1 and VEGF protein expression (Fig. 5D, E). In the tube-forming assay using C166 cells, the δ-TT-BMSC CM group had more extensive endothelial tubules and more complex networks connecting cell clusters compared to the other two groups (Fig. 5F). These findings demonstrated that δ-TT could promote the angiogenesis ability of BMSCs.

### δ-TT-BMSCs enhanced PAM-212 cell activity

**Fig. 6:**
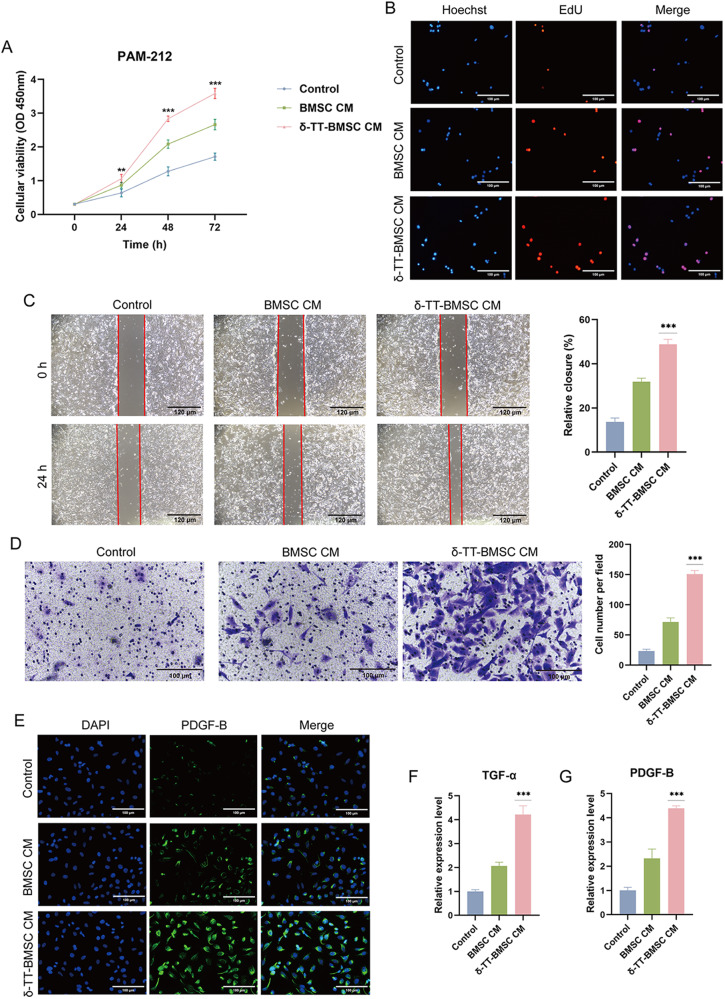
The effect of δ-TT-BMSCs on PAM-212 cells. **A** CCK8 experimental results showed cell proliferation in three groups. **B** EDU staining was utilized to detect cell proliferation (magnification: ×200, Bar = 100 μm). **C** Scratch assay indicated the migration abilities of PAM-212 cells in control, BMSC CM, and δ-TT-BMSC CM groups for 24 h, respectively (magnification: ×100, Bar = 120 μm). **D** The transwell migration experiment showed the transmigrated PAM-212 cells (magnification: ×200, Bar = 100 μm). **E** IF results showed the PDGF-B expression (magnification: ×200, Bar = 100 μm). **F**, **G** qRT-PCR was used to detect the expression of TGF-α and PDGF-B in PAM-212 cells treated with different CM. **p < 0.01, ***p < 0.001.

The proliferation capacity of PAM-212 cells was increased in the δ-TT-BMSC CM group (Fig. 6A, B). There were significantly more migrating cells in the inward scratch zone in the δ-TT-BMSC CM group than in the other two groups (Fig. 6C). The migration cell number in the down chamber was the highest in the δ-TT-BMSC CM group, followed by the BMSC CM group and control group (Fig. 6D). Keratinocytes release the growth factors PDGF-B and TGF-α promoting wound healing throughout the healing process [20]. The number of PDGF-B-positive cells was dramatically increased in the δ-TT-BMSC CM group (Fig. 6E). In addition, the δ-TT-BMSC CM group outperformed the other two groups in terms of promoting TGF-α and PDGF-B expression (Fig. 6F, G). These findings suggested that δ-TT-BMSCs showed positive regulation on keratinocytes with promoted cell proliferation, migration, and related factor secretion.

### δ-TT-BMSCs increased NIH-3T3 cell activity

**Fig. 7:**
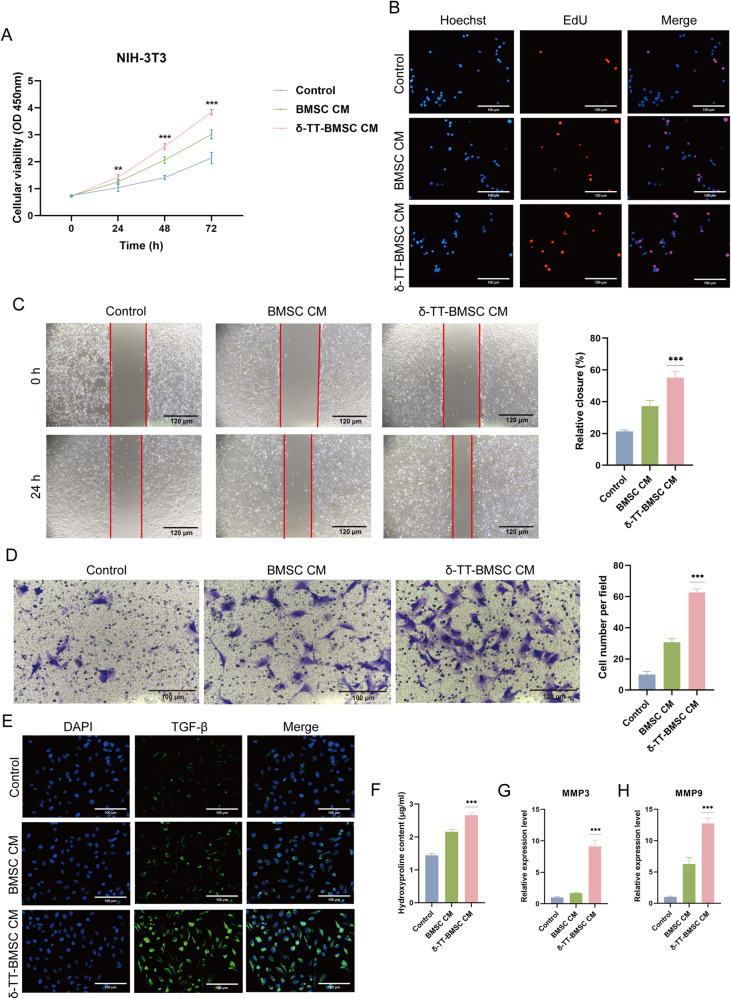
The effect of δ-TT-BMSCs on NIH-3T3 cells. **A** CCK8 results showed cell proliferation in three groups. **B** EDU staining was utilized to detect cell proliferation (magnification: ×200, Bar = 100 μm). **C** Scratch assay indicated the migration abilities of NIH-3T3 cells in control, BMSC CM, and δ-TT-BMSC CM groups for 24 h, respectively (magnification: ×100, Bar = 120 μm). **D** The transwell migration experiment showed the transmigrated NIH-3T3 cells (magnification: ×200, Bar = 100 μm). **E** IF results showed the TGF-β expression (magnification: ×200, Bar = 100 μm). **F** The different hydroxyproline content of NIH-3T3 cells in three groups. **G**, **H** qRT-PCR was used to detect the expression of MMP-3 and MMP-9 in NIH-3T3 cells treated with different CM. **p < 0.01, ***p < 0.001.

The proliferation capacity of NIH-3T3 cells was significantly increased in the δ-TT-BMSC CM group compared with the other groups (Fig. 7A, B). In addition, the δ-TT-BMSC CM group exhibited a greater capacity for migration and invasion (Fig. 7C, D). The TGF-β-positive cells increased dramatically in the δ-TT-BMSC CM group (Fig. 7E). Furthermore, the hydroxyproline content, an essential component of collagen synthesis, was higher in the δ-TT-BMSC CM group than in the control and BMSC CM groups (Fig. 7F). Moreover, the expressions of MMP-3 and MMP-9 were also higher in the δ-TT-BMSC CM group (Fig. 7G, H). Thus, δ-TT-BMSCs possessed a stronger ability to promote NIH-3T3 cell proliferation migration and collagen synthesis.

### δ-TT-BMSCs inhibited ferroptosis in both PAM-212 and NIH-3T3 cells

**Fig. 8:**
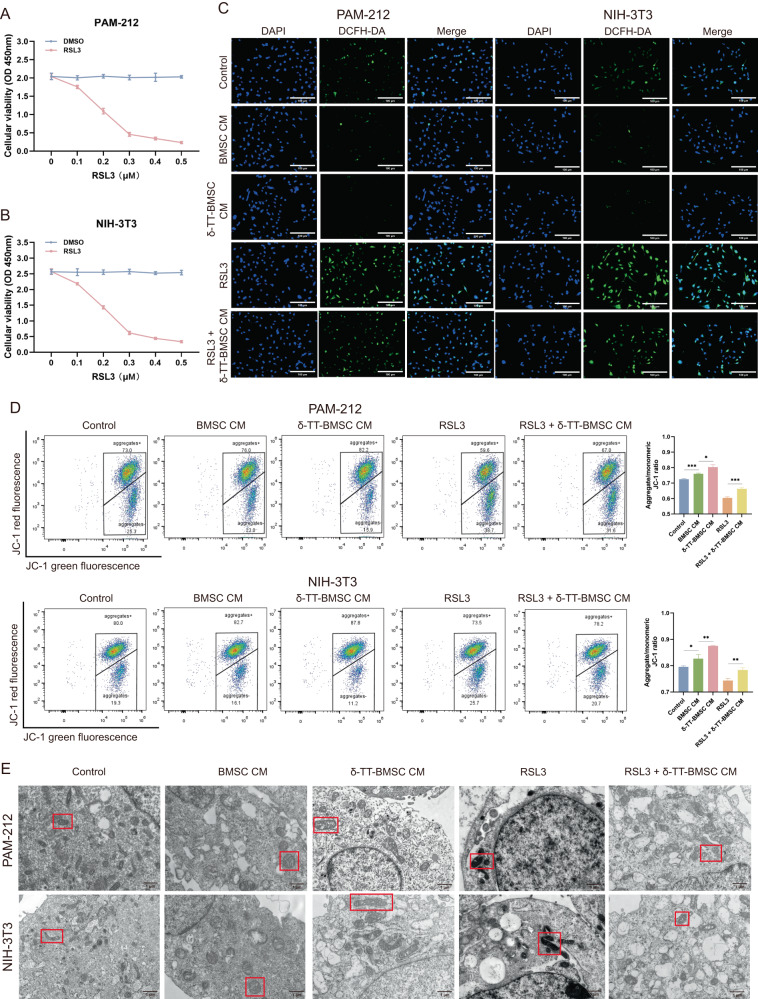
δ-TT-BMSCs inhibited ferroptosis in vitro. **A**, **B** Screening the optimum amount of RSL3 to treat PAM-212 and NIH-3T3 cells. **C** The intracellular ROS of PAM-212 and NIH-3T3 cells with different treatments (magnification: ×200, Bar = 100 μm). **D** FCM results showed the different mitochondrial membrane potentials in five groups. **E** Representative images obtained by electron microscopy. The red box indicated mitochondria (magnification: ×5000, Bar = 1 μm). *p < 0.05, **p < 0.01, ***p < 0.001.

RSL3, GPX4 inhibitor, significantly decreased proliferation of PAM-212 and NIH-3T3 cells, as determined by the CCK8 assay (Fig. 8A, B). The DCFH-DA method confirmed that RSL-induced ROS accumulation could be attenuated by δ-TT-BMSC CM (Fig. 8C). The FCM analysis showed that δ-TT-BMSC CM also reversed the RSL3-induced decrease in mitochondrial membrane potential (Fig. 8D). The TEM observation proved that the ferroptosis-related morphological features, including mitochondria with degenerating cristae, were reversed in δ-TT-BMSC CM (Fig. 8E).

**Fig. 9:**
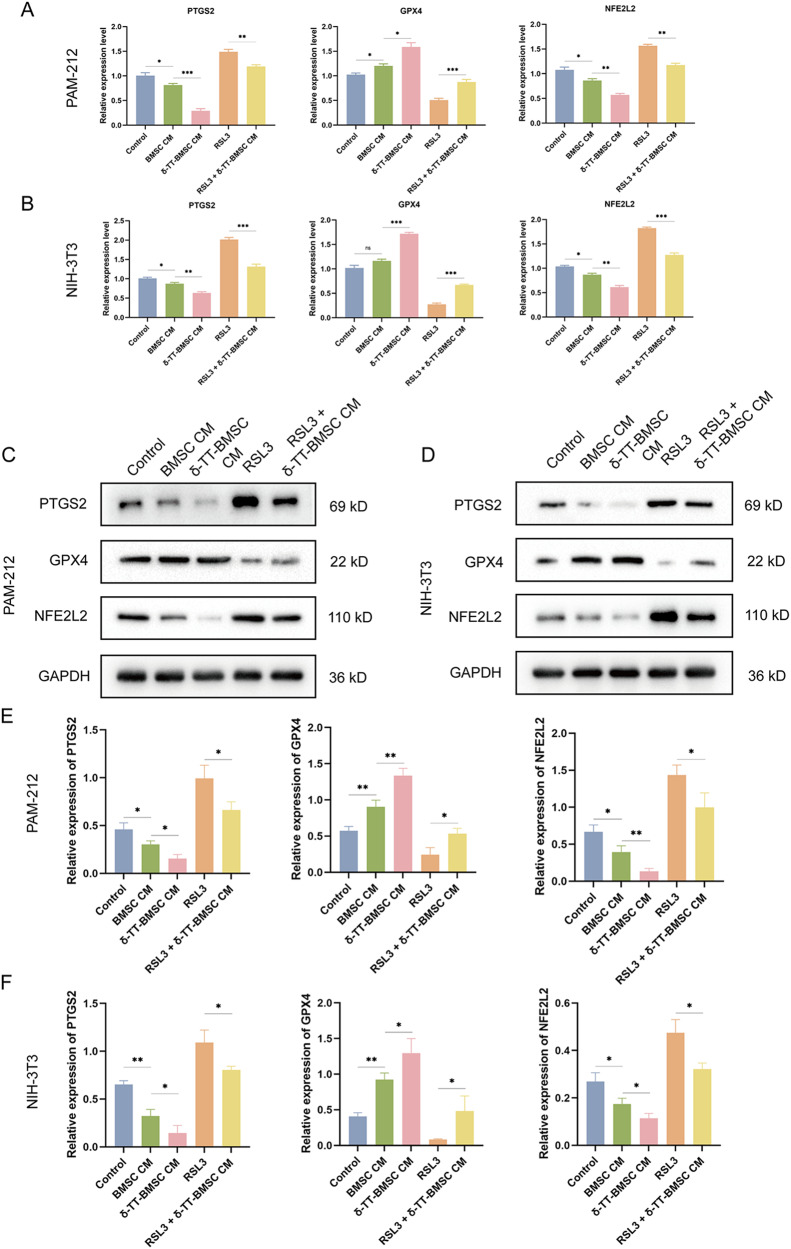
Using ferroptosis marker genes to identify the ability of δ-TT-BMSCs to inhibit ferroptosis. **A**, **B** qRT-PCR was used to detect the expression of PTGS2, GPX4 and NFE2L2 in PAM-212 and NIH-3T3 cells with different treatments. **C**–**F** The western blot results of PTGS2, GPX4, and NFE2L2 protein in PAM-212 and NIH-3T3 cells, and related quantitative analysis. *p < 0.05, **p < 0.01, ***p < 0.001.

RT-PCR results showed that PTGS2 and NFE2L2 expressions were down-regulated in the δ-TT-BMSC CM group, and significantly increased in the RSL3 group, which was reversed after δ-TT-BMSC CM preconditioning. However, GPX4 expression was up-regulated in the δ-TT-BMSC CM group and significantly decreased in the RSL3 group and could be reversed with δ-TT-BMSC CM preconditioning (Fig. 9A, B). The results of the WB assay were consistent with the RT-PCR results (Fig. 9C–F). These results suggested that δ-TT-BMSC CM could inhibit ferroptosis and rescue RSL3-induced ferroptosis in PAM-212 and NIH-3T3 cells.

### BACH1 was positively correlated with ferroptosis in both PAM-212 and NIH-3T3 cells

**Fig. 10:**
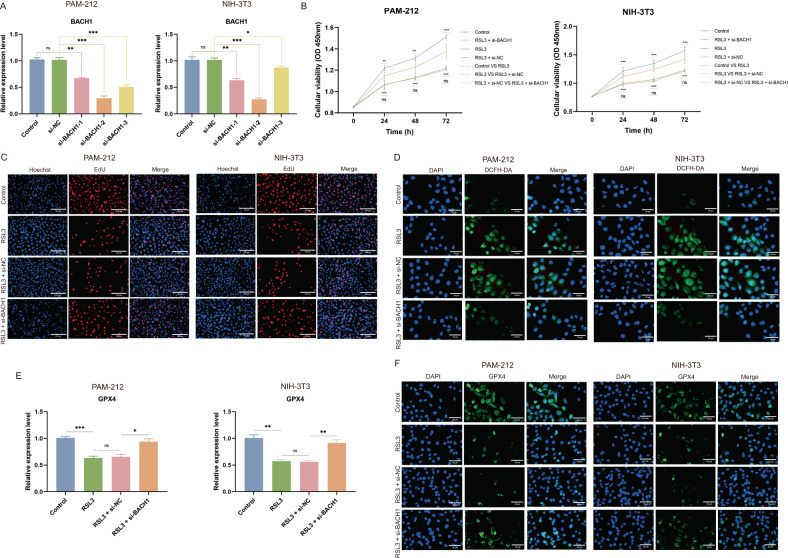
The correlation of BACH1 and ferroptosis in vitro. **A** qRT-PCR was used to detect the silencing efficiency of BACH1 in PAM-212 and NIH-3T3 cells. **B** CCK8 results showed cell proliferation in four groups. **C** EDU staining was utilized to detect cell proliferation (magnification: ×200, Bar = 100 μm). **D** The intracellular ROS of PAM-212 and NIH-3T3 cells with different treatments (magnification: ×400, Bar = 50 μm). **E**, **F** The expression of GPX4 in PAM-212 and NIH-3T3 cells with different treatments (magnification: ×400, Bar = 50 μm). *p < 0.05, **p < 0.01, ***p < 0.001.

To further explore the role of BACH1 in ferroptosis, we examined the effect of the BACH1 gene on cell proliferation and expression of the ferroptosis marker gene. Si-BACH1-2 successfully knocked down the expression of BACH1 in PAM-212 and NIH-3T3 cells (Fig. 10A). The proliferation ability of both PAM-212 and NIH-3T3 cells in the RSL3 group was suppressed compared with the control group. However, the depletion of BACH1 could reverse the RSL3-induced decrease in cell proliferation (Fig. 10B, C). Moreover, the DCFH-DA assay confirmed that RSL3-induced ROS accumulation could be attenuated by the depletion of BACH1 (Fig. 10D). The expression of GPX4 was decreased by RSL3 and could be reversed by the depletion of BACH1 (Fig. 10E, F). These results suggested that BACH1 was positively correlated with ferroptosis and that the depletion of BACH1 could reverse the RSL3-induced ferroptosis.

### δ-TT-BMSCs inhibited BACH1-related ferroptosis and activated PI3K/AKT signaling pathway

**Fig. 11:**
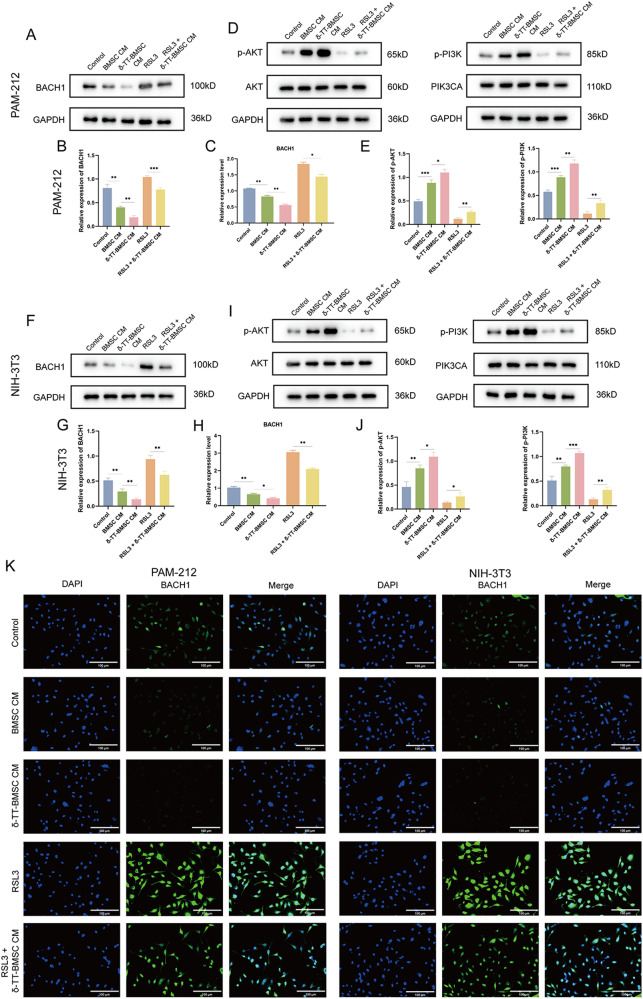
The mechanism of δ-TT-BMSCs inhibiting ferroptosis in vitro. **A**, **B** The western blot results of BACH1 protein in PAM-212 cells and related quantitative analysis. **C** qRT-PCR was used to detect the expression of BACH1 in PAM-212 cells. **D**, **E** Western blot analysis showed the AKT, p-AKT, PIK3CA, and p-PI3K protein expression in PAM-212 cells. **F**, **G** The western blot results of BACH1 protein in NIH-3T3 cells and related quantitative analysis. **H** qRT-PCR was used to detect the expression of BACH1 in NIH-3T3 cells. **I**, **J** Western blot analysis showed the AKT, p-AKT, PIK3CA, and p-PI3K protein expression in NIH-3T3 cells. **K** IF results showed the BACH1 expression in PAM-212 and NIH-3T3 cells (magnification: ×200, Bar = 100 μm). *p < 0.05, **p < 0.01, ***p < 0.001.

The expression levels of BACH1 and the activation of PI3K/AKT pathway in PAM-212 and NIH-3T3 cells were assessed using WB. The results showed that the expression of BACH1 was significantly reduced in the δ-TT-BMSC CM group and increased in the RSL3 group compared to the control group, which was reversed by δ-TT-BMSC CM treatment (Fig. 11A, B, F, G). The same results were demonstrated by RT-PCR (Fig. 11C, H) and IF (Fig. 11K). Meanwhile, the expression levels of p-AKT and p-PI3K were significantly higher in the δ-TT-BMSC CM group compared to the control group, while the expression of p-Akt and p-PI3K was significantly lower in the RSL3 group and could be reversed by δ-TT-BMSC CM (Fig. 11D, E, I, J). These results suggested that δ-TT-BMSC CM might inhibit ferroptosis by down-regulating the expression of BACH1 and activating PI3K/AKT signaling pathway

### δ-TT-BMSCs improved wound healing in vivo

**Fig. 12:**
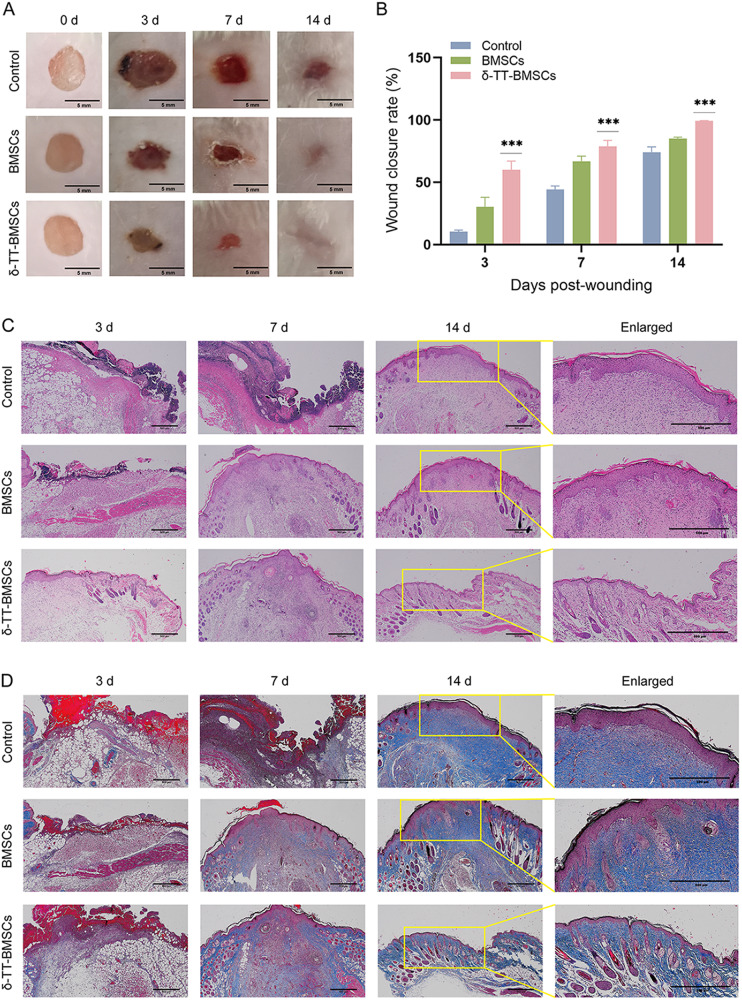
δ-TT-BMSCs improved wound healing in mice. **A** A summary of the differences in the size of the wound created in the back skin of mice among the three groups on day 0, 3, 7, and 14 following treatment (Bar = 5 mm). **B** The rate of wound healing following engraftment of BMSCs or δ-TT-BMSCs. **C** HE staining of wound granulation tissue on day 3, 7, and 14 following wounding in the groups of control, BMSC, and δ-TT-BMSC (magnification: ×40, Bar = 500 μm). **D** Masson staining of repaired tissue on day 3, 7, and 14 in control, BMSC, and AA2G-treated BMSC groups (magnification: ×40, Bar = 500 μm). ***p < 0.001.

Compared to the control group, the wound healing rate was accelerated in mice treated with BMSCs on day 3, 7, and 14. However, the wound healing rate in the BMSCs group was inferior to that in the δ-TT-BMSC group, with a statistically significant difference (Fig. 12A, B). Similarly, HE staining revealed that the δ-TT-BMSC group had a considerably higher cell density and epithelialization of granulation tissue compared to the other two groups (Fig. 12C). In addition, both the BMSC group and the δ-TT-BMSC group had more organized collagen deposition in the wound tissue, but the δ-TT-BMSC group had a greater collagen density (Fig. 12D). These findings showed that δ-TT-BMSCs might hasten wound healing by promoting epithelialization and increasing collagen production.

### δ-TT-BMSCs inhibited ferroptosis in a wound model

**Fig. 13:**
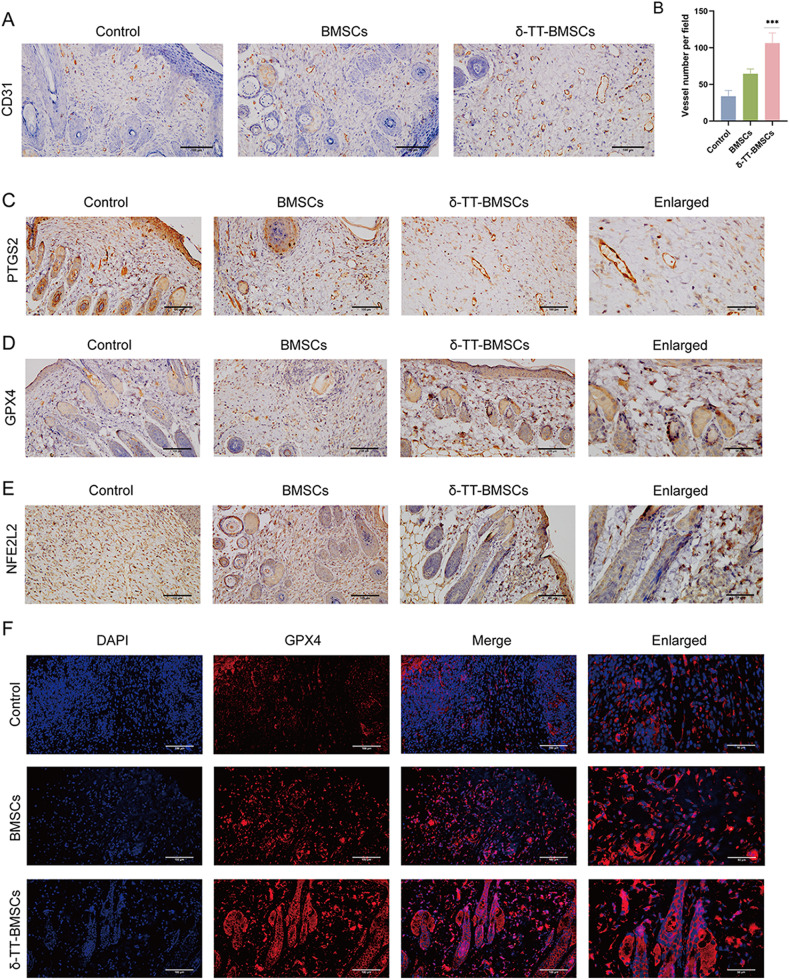
δ-TT-BMSCs promoted angiogenesis and inhibited ferroptosis in wound tissues. **A** Typical pictures of the wound tissue stained with CD31 in control, BMSC, and δ-TT-BMSC groups (magnification: ×200, Bar = 100 μm). **B** The microvessel density in the wound was evaluated by CD31-positive staining, and the number of CD31-positive microvessels in each visual field was calculated. **C**–**E** IHC results showed the PTGS2, GPX4, and NFE2L2 expression in control, BMSC, and δ-TT-BMSC groups on day 14 (magnification: ×200, Bar = 100 μm). **F** IF results showed the GPX4 expression in control, BMSC, and δ-TT-BMSC groups on day 14 (magnification: ×200, Bar = 100 μm). ***p < 0.001.

**Fig. 14:**
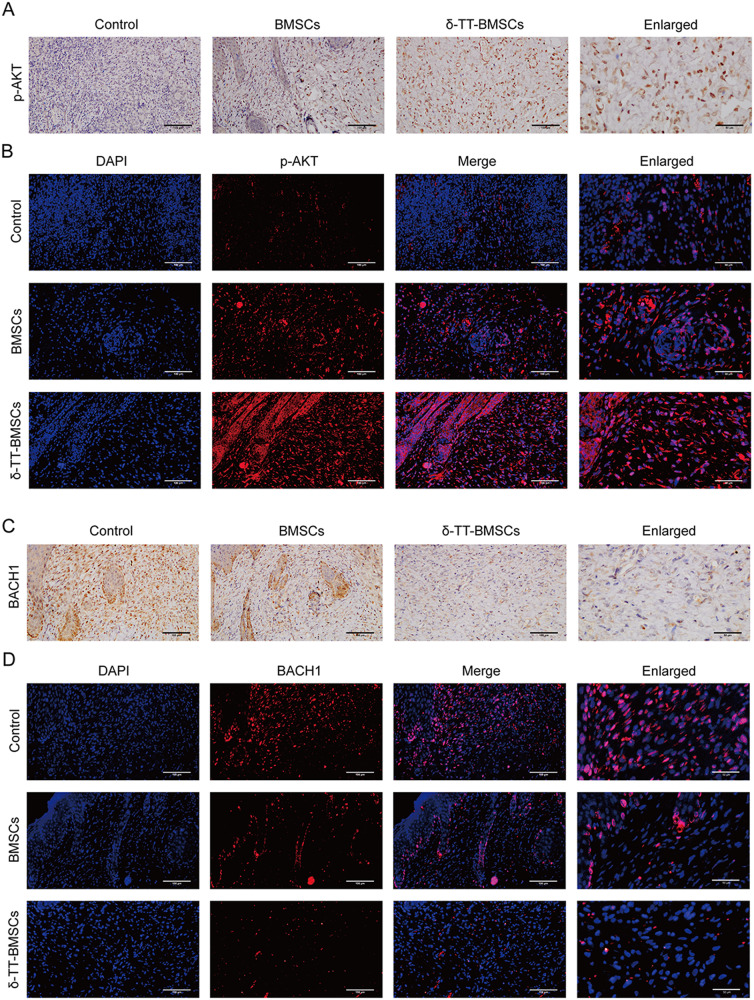
δ-TT-BMSCs down-regulated the BACH1 expression and activated PI3K/AKT signaling pathway in wound tissues. **A**, **B** IHC and IF results showed the p-AKT expression in control, BMSC, and δ-TT-BMSC groups on day 14 (magnification: ×200, Bar = 100 μm). **C**, **D** IHC and IF results showed the BACH1 expression in control, BMSC, and δ-TT-BMSC groups on day 14 (magnification: ×200, Bar = 100 μm).

The results of IHC staining demonstrated an increase of neovascularization in the wound tissue after BMSC treatment, with the lowest number of new blood vessels in the control group. The greatest increase in blood vessels was seen in the δ-TT-BMSC group, further supporting the enhanced angiogenic potential of δ-TT-BMSCs (Fig. 13A, B). IHC and IF results showed that GPX4 expression was up-regulated (Fig. 13D, F) while PTGS2 and NFE2L2 expression was down-regulated (Fig. 13C, E) in the δ-TT-BMSC group compared to the control group, suggesting that δ-TT-BMSCs could effectively inhibit the development of wound ferroptosis. At the same time, a higher number of p-AKT positive cells (Fig. 14A, B) and fewer BACH1 positive cells (Fig. 14C, D) were observed in the new wound area of the δ-TT-BMSC group, demonstrating the involvement of the BACH1 and AKT pathway in wound healing of δ-TT-BMSC group.

## Discussion

Nowadays, local stem cell transplantation has become a desirable approach for accelerating wound healing due to its ability to stimulate the release of growth factors [21]. According to our study, down-regulating BACH1 expression and activating the PI3K/AKT pathway, which prevented ferroptosis, allowed δ-TT-BMSCs to considerably speed up wound healing in mice as well as enhance collagen deposition and angiogenesis at the wound site. By boosting the paracrine function of BMSCs, δ-TT therapy might also positively regulate keratinocytes, fibroblasts, and vascular endothelial cells in vitro. As a result, δ-TT-BMSCs had a higher therapeutic potential for wound healing, particularly in terms of inhibiting ferroptosis.

The wound healing process is composed of four interrelated phases: Hemostasis, inflammation, proliferation, and tissue remodeling [22]. During this process, ROS are produced by activated macrophages as organic byproducts of oxygen metabolism [23]. To minimize ROS-induced cellular damage during tissue repair, it’s crucial to control oxidative stress and inflammatory response [24]. Vitamin E is the main fat-soluble antioxidant in the skin and consists of eight compounds divided into two groups: TPs, and TTs [25]. Vitamin E is mostly known for its role as an antioxidant because it defends cell membranes and polyunsaturated lipids from ROS assault by stimulating the activation of several signal transduction pathways [26]. δ-TT, a vitamin E derivative, is valuable for its strong anti-oxidant activity and anticancer effects [17, 18]. According to Shen et al., δ-TT has the potential to treat nasopharyngeal carcinoma by inducing apoptosis in CNE3 nasopharyngeal carcinoma cells through the caspase-3 signaling [27]. TTs have been shown by Li et al. to delay the onset of atherosclerosis in both human patients and preclinical animal models [28]. However, the application of δ-TT in wounds has been less studied. Hoff et al. discovered that the garcinoic acid, a δ-TT derivative, enhanced wound healing and the quality of newly generated tissue in a mouse model with splint wounds [18]. In addition, Casati et al. verified that δ-TT might improve BMSC recruitment and encourage MC3T3-E1 differentiation and migratory behavior [19]. Therefore, we conducted in vivo and in vitro experiments to evaluate the properties of δ-TT in wound healing. The results showed that δ-TT-BMSCs promoted fibroblast proliferation, migration, angiogenesis, keratinocyte proliferation, migration, and pro-wound healing capacity. These findings were further supported by consistent results from animal experiments, highlighting the crucial role of δ-TT-BMSCs in wound healing.

Ferroptosis is an iron-dependent cell death. Li et al. identified characteristic changes associated with ferroptosis in a diabetic rat model, where the ferroptosis inhibitor Fer-1 accelerated wound healing by activating the PI3K/AKT signaling pathway [12]. By the bioinformatic analysis, we found that wound ferroptosis was more prevalent and might be an important factor affecting wound healing. The heatmap of the ferroptosis driver gene showed that BACH1 expression was positively correlated with ferroptosis and was highly expressed in the wound group. BACH1 is a transcription factor that regulates a variety of physiological processes including heme homeostasis, oxidative stress response, senescence, cell cycle, and mitosis [29]. Su et al. showed that the Nrf2-induced miR-23a-27a-24-2 cluster promoted intestinal mucosal damage repair by targeting the BACH1/HO-1 axis in inflammatory bowel disease [30]. Jian et al. demonstrated that hemoglobin alleviated hyperoxia-induced inhibition of VEGF activity in human microvascular endothelial cells (HMEC-1) by inhibiting BACH1. The inhibition of BACH1 could promote the proliferation, migration, and angiogenesis of HMEC-1 cells [31]. However, few studies have focused on the mechanism of BACH1 in wound healing. In our mouse wound model, BACH1 was highly expressed in wound tissue, foreshadowing a positive correlation with ferroptosis. There is a close relationship between BACH1 and ferroptosis, as evidenced by the research of Nishizawa et al., who verified that BACH1 regulated cellular differentiation and lipid metabolism to control ferroptosis, and could be used to achieve anti-cancer cell therapy in the future [32]. The study by Namgaladze revealed that silencing BACH1 decreased unstable iron pools and lipid peroxidation while increasing macrophage resistance to ferroptosis [33]. In our experiments, δ-TT-BMSC CM significantly inhibited ferroptosis in PAM-212 and NIH-3T3 cells and rescued RSL3-induced ferroptosis, probably by inhibiting BACH1 expression.

A variety of synergistic signaling pathways are initiated in the wound setting, of which the PI3K/AKT signaling pathway is one of the more intensively studied. For example, Guo et al. demonstrated that preconditioning BMSCs with H2O2 enhances their proliferation, migration, and survival through activation of the PI3K/AKT/mTOR pathway, thereby increasing their therapeutic potential in wound healing [34]. In addition, Wang et al. found that hypoxic adipose stem cell-derived exosomes promoted high-quality diabetic wound healing by activating the PI3K/AKT signaling pathway [35]. Our study supports these findings by showing that the δ-TT-BMSCs promote wound healing through activation of the PI3K/AKT signaling pathway. In vivo, experiments demonstrated that PI3K/AKT signaling pathway was significantly activated in the δ-TT-BMSC CM group. Furthermore, our in vitro experiments revealed that the δ-TT-BMSC group exhibits the best wound healing outcomes, with the highest expression of p-AKT seen through IHC and IF results.

Meanwhile, this study still has some limitations. Firstly, further research in subsequent studies is needed to determine whether locally injected BMSCs can migrate to distant sites or attract host-derived stem cells. Secondly, the δ-TT-BMSC CM has a complicated composition. Further research is therefore needed to determine which specific factors or features of the composition inhibit ferroptosis and promote wound healing. In addition, since many different types of cells are involved in wound healing, it is important to further investigate whether other cells also undergo ferroptosis and whether this also has an impact on the wound surface. Although the preliminary bioinformatic analysis indicates that keratinocytes and fibroblasts are more likely to occur ferroptosis and enrich in the PI3K/AKT signaling pathway. But other cells such as vascular endothelial cells are also significant players in wound healing. The results of the bioinformatic analysis may be skewed by the sample size, and further sequencing data is required to confirm these findings. The inflammatory response is also a crucial component of wound healing. The local immune regulation of the wound by δ-TT-BMSC CM is interesting to study, particularly with ferroptosis. It would be intriguing to find out if other programmed cell death pathways (PCDs), such as apoptosis, cell necrosis, and autophagy, also play a significant role in wound healing. Finally, the wound-healing process is not identical in mice and humans. Large-scale clinical trials are needed to further validate and optimize the concentration and administration method of δ-TT-BMSCs for clinical use in wound healing.

## Conclusions

**Fig. 15:**
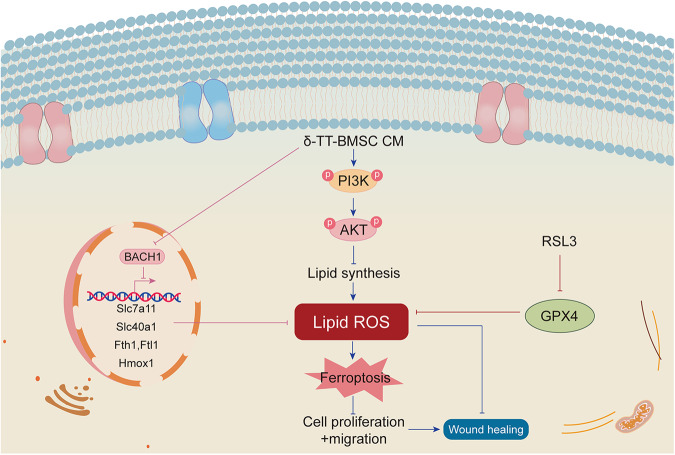
Schematic diagram of the mechanism of δ-TT-BMSCs inhibiting ferroptosis to repair the wound. By inhibiting the transcription of erastin-induced protective genes, BACH1 promotes ferroptosis. These genes, which include Hmox1, Slc7a11, Fth1, Ftl1, and Slc40a1, were related to the production of GSH and the intracellular metabolism of labile iron. RSL3 is a ferroptosis inducer and works by preventing the expression of GPX4. δ-TT-BMSCs reduce lipid peroxidation damage by down-regulating BACH1 expression and activating the PI3K/AKT signaling pathway to inhibit ferroptosis, thereby effectively improving wound healing.

In summary, our findings imply that δ-TT can stimulate BMSC proliferation, angiogenesis, and migration in vitro, promoting wound healing in mice and facilitating angiogenesis and collagen deposition, most likely by inhibiting BACH1 expression and activating the PI3K/AKT signaling pathway (Fig. 15). Consequently, δ-TT is a crucial regulator that strengthens the beneficial effects of BMSCs in wound healing.

## Materials and methods

### Transcriptome data mining

All microarray data were downloaded from the Gene Expression Omnibus (GEO) database (https://www.ncbi.nlm.nih.gov/geo/query/acc.cgi?acc=GSE23006). The raw data were downloaded and series matrix files were used to further analysis by R software. We performed different analyses by selecting the 12 h post-wounding sample as treated by the limma package. The R software ggord package was used to draw the PCA plot. The ComplexHeatmap package (1.19.1) was used for the visualization of heatmaps and complex summary plots [36]. The R package ggplot2 was used to draw the volcano plot. Kyoto Encyclopedia of Genes and Genomes (KEGG) pathway analysis and Gene Ontology (GO) analysis were performed using the clusterProfiler R package (v4.0.0) [37]. FerrDb was the world’s first manually curated database for ferroptosis regulators and ferroptosis-disease associations from published journal articles. Ferroptosis marker genes were derived from FerrDb (http://www.zhounan.org/ferrdb/current/).

### Single-cell data mining

The GEO database was used to download the single-cell sequencing dataset GSE153596, and the raw data included single-cell samples of both wound and unwounded tissues. Illumina HiSeq 4000 (Mus musculus) served as the sequencing platform. Using the Seurat package, raw data from GSE153596 were transformed into expression matrices and preprocessed. The data were first subjected to quality control using the criteria that each gene was found in more than 95% of the samples and that the average number of reads per sample was >10,000. There were 8887 cells from wound tissue and 2315 cells from unwounded tissue in the filtered data set. The NormalizeData method’s default parameters were then used to perform normalization. Finally, the cells were clustered using the t-SNE algorithm for dimensionality reduction. The SingleR package was used to perform cell type annotation on the preprocessed single-cell data, and the built-in MouseRNAseqData dataset served as the reference dataset. The expression of prostaglandin-endoperoxide synthase 2 (PTGS2), glutathione peroxidase 4 (GPX4), and nuclear factor, erythroid 2 like 2 (NFE2L2) was compared in the wound and unwounded tissues at the single-cell level. Statistical tests were run using the t-test method, and box plots were created for visualization. Stack plots were utilized to display the percentage of cellular abundance in the wound and unwound tissues, which was plotted using the ggplot2 package. The pheatmap package was used to show the connection between the three levels of gene expression and phenotype. Using the limma package, the differentially expressed genes were screened at the single cell level using the criteria of |log2FC | > 0.5 and adjusted *P* value < 0.05. GO and KEGG enrichment analysis of the differentially expressed genes was performed using clusterProfiler, and the pathways with *P* value < 0.05 were identified as significantly enriched pathways.

### Isolation, culture, and identification of mouse BMSCs

BALB/C mice (3 w, male) were dislocated and then immersed in 75% alcohol for 15 min [34]. The bone marrow cavity was then rinsed with a culture medium containing Dulbecco’s Modified Eagle Medium/Nutrient Mixture F-12 (DMEM/F12) (Gibco, USA), and the bone marrow cell suspension was obtained under sterile conditions. DMEM/F12, 20% fetal bovine serum (FBS, Gibco, USA), 100 U/mL penicillin, and 100 μg/mL streptomycin were added to culture flasks after single cells were centrifuged and filtered through a 200 mesh filter. At 37 °C and saturated CO_2_ humidity, cells were set up in a static culture. BMSCs on passage 3 were prepared, and treated with anti-CD29, CD44, CD90, CD31, CD34, and CD105 antibodies (Becton Dickinson, USA), and then incubated in the dark for 30 min. Following PBS washing, samples were suspended and examined by flow cytometry (FCM) (Becton Dickinson, USA). For the ensuing experiments, 3rd–7th generation BMSCs were gathered. The system was DMEM/F12 with 10% fetal bovine serum, 0.5 μM dexamethasone, 50 μM isobutylmethylxanthine (IBMX), 10 μg/mL insulin, and 50 μM indomethacin, and was used to culture BMSCs for 28 d to induce their adipogenesis [38]. To stimulate osteogenesis and chondrogenesis, BMSCs were cultured in mesenchymal stem cell (MSC) osteogenic differentiation medium and MSC chondrogenic differentiation medium (Cyagen Biosciences, USA).

### Establishment of mouse wound healing model

A sodium pentobarbital injection of 60 mg/kg was used to systematically anesthetize 45 BALB/C mice (8 w, male). A full-thickness skin wound model was made on the mouse back using an 8 mm diameter biopsy punch (Kai Medical, Solingen, Germany) after dehairing treatment. Mice were randomly divided into three groups: control group (100 μL PBS, *n* = 15), BMSC group (2 × 10^6^ BMSCs resuspended in 100 μL PBS, *n* = 15), and δ-TT-BMSC group (2 × 10^6^ δ-TT-BMSCs resuspended in 100 μL PBS, *n* = 15). Depending on the group, PBS or cell suspensions were injected at 4 points around the wound and 1 point in the wound center. On day 0, 3, 7, and 14 following treatment, wound closure areas were photographed and tissue samples were collected. Images of skin wounds were imported into the Image J software (National Institutes of Health, USA) to analyze wound healing. This study was approved by the Ethics Committee of Animal Experimental Research Center of Zhejiang Chinese Medical University.

### Cell culture, proliferation and migration

Mouse keratinocytes PAM-212 were cultured in 90% RPMI 1640 medium (Gibco, USA) and 10% fetal bovine serum, whereas mouse embryonic fibroblasts NIH-3T3 and mouse vascular endothelial cells C166 were grown in 90% DMEM (Gibco, USA) and 10% fetal bovine serum. By using the CCK-8 test, the ideal concentration of BMSCs preconditioning with δ-TT (Rhawn, China) was shown to be 10 μg/mL, which was consistent with the findings of Casati [19]. BMSCs were cultivated for 1 d in the medium containing 10 μg/mL δ-TT and subsequently for 1 d in the serum-free medium, and the conditioned medium (CM) obtained was named δ-TT-BMSC CM. BMSCs were also cultured in the serum-free medium for 1 d and CM was collected. After being exposed to 2 distinct CMs for 24 h, cell growth, migration, and collagen production were observed in both PAM-212 and NIH-3T3 cells. As for the tube formation, the substrate (BD Biosciences) was thawed and transferred to 24-well culture plates for solidification. BMSC CM or δ-TT-BMSC CM were applied to C166 cells for 24 h at 37 °C. Tube formation was observed with a phase-contrast microscope (Olympus Corporation, Tokyo, Japan).

After 24 h of δ-TT treatment, the BMSCs were rinsed, fixed in 70% ethanol at 4 °C, and resuspended in PBS. Following the addition of 400 μL propidium iodide (Sigma-Aldrich, USA) for 30 min, FCM was used to analyze the G1, S, and G2 cell cycles. In addition, cell proliferation tests were carried out following the guidelines for the EDU staining kit (Beyotime, China), and the CCK8 kit (Yeasen, China). As mentioned above, the PAM-212 and NIH-3T3 proliferation assays were conducted.

BMSCs were divided into the control and δ-TT-BMSC groups and inoculated in 6-well plates. When the cells had reached 90% of the confluence, a scratch was made via a 1 mL microtube tip, and the serum-free medium was replaced. Following the taking of photographs at 0 and 24 h after the injury, the wound width was measured using AI software to determine the wound closure rate. The PAM-212 and NIH-3T3 cells were divided into the control, BMSC CM, and δ-TT-BMSC CM groups, and the scratch assay was carried out as previously described. Boyden chambers (Corning Costar, USA) with an 8-μm pore size were used for transwell migration assays. BMSCs were put in the upper Boyden chamber, and 10 μg/mL δ-TT was added to the lower chamber. PAM-212 and NIH-3T3 cells were similarly added to the upper Boyden chamber, and two CM were separately added to the lower chamber. After 24 h culture, the submembrane migrating cells were fixed in 4% paraformaldehyde for 15 min and stained with 0.5% crystalline violet. Migrating cells were observed and were counted under a SOPTOP CX40 microscope (Shanghai, China).

### Cell transfection, RNA extraction, and qRT-PCR

The synthesized small interfering RNA (siRNA) was dissolved in solution at a concentration of 20 μmol/L. PAM-212 and NIH-3T3 cells were seeded in 6-well plates at a density of 4 × 10^5^/mL for 24 h. According to the reagent instructions, siRNA was transfected into cells by Lipofectamine 3000 (Invitrogen, Carlsbad, CA, USA). After 24 h of transfection, the silencing efficiency was tested in subsequent experiments. All siRNA sequences for BACH1 are available in Table S1. Total RNA was extracted from the cell samples via Trizol Reagent (Invitrogen). Using a high-volume cDNA reverse transcription kit (Vazyme, China) under the manufacturer’s instructions, stable cDNA was obtained. SYBR Green PCR Master Mix (Vazyme China) was used for qRT-PCR, with GAPDH serving as an internal control. All primer sequences were shown in Table S2.

### Western blot (WB)

Cells were divided into five groups: control, BMSC CM, δ-TT-BMSC CM, RSL3, and RSL3 + δ-TT-BMSC CM groups. Cells were gathered and suspended in cell lysis buffer (Cell Signaling Technology, USA) after 24 h treatment, and the supernatant was harvested by centrifugation. Samples were electroblotted onto polyvinylidene fluoride (PVDF) membranes (Bio-Rad, USA) after being separated on a 10% polyacrylamide-SDS gel. PVDF membranes were then blotted with the primary antibody at 4 °C overnight after being blocked in 5% skimmed milk in tris-buffered saline with Tween 20 (TBST) for 1 h. After being washed, PVDF membranes were incubated for 3 h at room temperature with HRP-coupled secondary antibodies (Proteintech, USA) at 1:5000. The ECL WB kit (Proteintech, USA) was used to detect protein signals, which were quantified by ImageLab software. The primary antibodies used in this study were described as follows: anti-VEGF antibody, anti-GAPDH antibody, anti-p-AKT antibody, anti-AKT antibody, anti-PTGS2 antibody, anti-NFE2L2 antibody, anti-BACH1 antibody (Proteintech, USA), anti-PIK3CA antibody, anti-p-PI3K antibody, anti-GPX4 antibody (Abclonal, China).

### Histological analysis

Normal and wound mouse skin samples were paraffin-embedded after being fixed in the 10% formalin solution. To observe wound re-epithelialization and collagen deposition, tissue sections were stained with hematoxylin and eosin (HE), and Masson trichrome respectively. Angiogenesis was assessed by using CD31 immunohistochemical (IHC) staining. Primary antibodies were applied to the sections before being incubated with peroxidase-conjugated secondary antibodies (Cell Signaling, MA). The staining color was visualized using the DAB Peroxidase Substrate Kit (Maxin, China) and was photographed using a SOPTOP CX40 microscope (Shanghai, China)

The following primary antibodies were utilized: anti-CD31 antibody, anti-GPX4 antibody, anti-NFE2L2 antibody, anti-PTGS2 antibody, anti-p-AKT antibody, and anti-BACH1 antibody (Proteintech, USA).

### Immunofluorescence (IF) staining

Cell samples and tissue sections were fixed in 4% paraformaldehyde. The samples were then sealed with a 2% (W/V) bovine serum albumin (BSA) solution before being incubated with the primary antibody at 4 °C overnight and the secondary antibody the next day at room temperature. DAPI nuclear dye was utilized to restain the samples. The following primary antibodies were utilized: anti-VEGF antibody, anti-TGF-β antibody, anti-p-AKT antibody, anti-BACH1 antibody (Proteintech, USA), anti-PDGF-B antibody, anti-GPX4 antibody (Abclonal, China).

### ROS assay

Following the manufacturer’s instructions, the 2′7′-dichlorodihydro-fluorescein diacetate (DCFH-DA) assay (Abcam Cambridge, UK) was used to measure intracellular ROS. In a nutshell, DCFH-DA (20 μM) was incubated with PAM-212 and NIH-3T3 cells in 96-well plates for 45 min at 37 °C in the dark. Photographs were taken using a fluorescent microscope (BX53, Olympus, Japan).

### Mitochondrial membrane potential assay

The mitochondrial membrane potential was detected via the fluorescent probe JC-1 (Invitrogen, CA, USA). In the normal membrane potential of mitochondria, JC-1 formed aggregates that emitted red fluorescence; in damaged and/or depolarized mitochondria, JC-1 formed monomers that emitted green fluorescence. According to the manufacturer’s instructions, cells were cultured at 37 °C in 6-well plates with 3 × 10^5^ cells per well. After 24 h, cells were stained with 5 µmol/L JC-1 at 37 °C for 15 min and were analyzed by FCM (Becton Dickinson, USA). Acquired data were analyzed using FlowJo software.

### Electron microscopy

The 6-well plate of cells was fixed for 24 h using a solution of 2.5% glutaraldehyde in PBS. The cells were first cleaned in 0.1 M PBS, then subjected to 0.1% Millipore-filtered cacodylate-buffered tannic acid treatment, 1% buffered osmium postfixation, and 1% Millipore-filtered uranyl acetate staining. Samples were dehydrated and embedded before being incubated for 24 h at 60 °C. The digital pictures were captured using a transmission electron microscope (HITACHI, Japan).

### Statistical analysis

With GraphPad Prism 8.0 (GraphPad, USA), the two-group analysis was conducted via the one-way analysis of variance with the *t*-test. To establish the statistical significance, the data were analyzed using the ANOVA with the posthoc analysis via Bonferroni/Dunn’s test for more than two groups. All experiments were implemented separately in triplicate. The threshold for statistical significance was set at *P* < 0.05.

### Supplementary information

Full and Uncropped Western Blots

Table S1

Table S2

## Data Availability

All datasets generated for this study are included in the paper and the supplementary materials.
