# Supplementary material for: δ-Tocotrienol preconditioning improves the capability of bone marrow-derived mesenchymal stem cells in promoting wound healing by inhibiting BACH1-related ferroptosis
Source: Cell Death Discov. 2023 Sep 22;9:349. doi: 10.1038/s41420-023-01653-1 (PMC10516898; doi:10.1038/s41420-023-01653-1)
Supplement: Supplementary file 1 — Full and Uncropped Western Blots [file 41420_2023_1653_MOESM1_ESM.pdf]

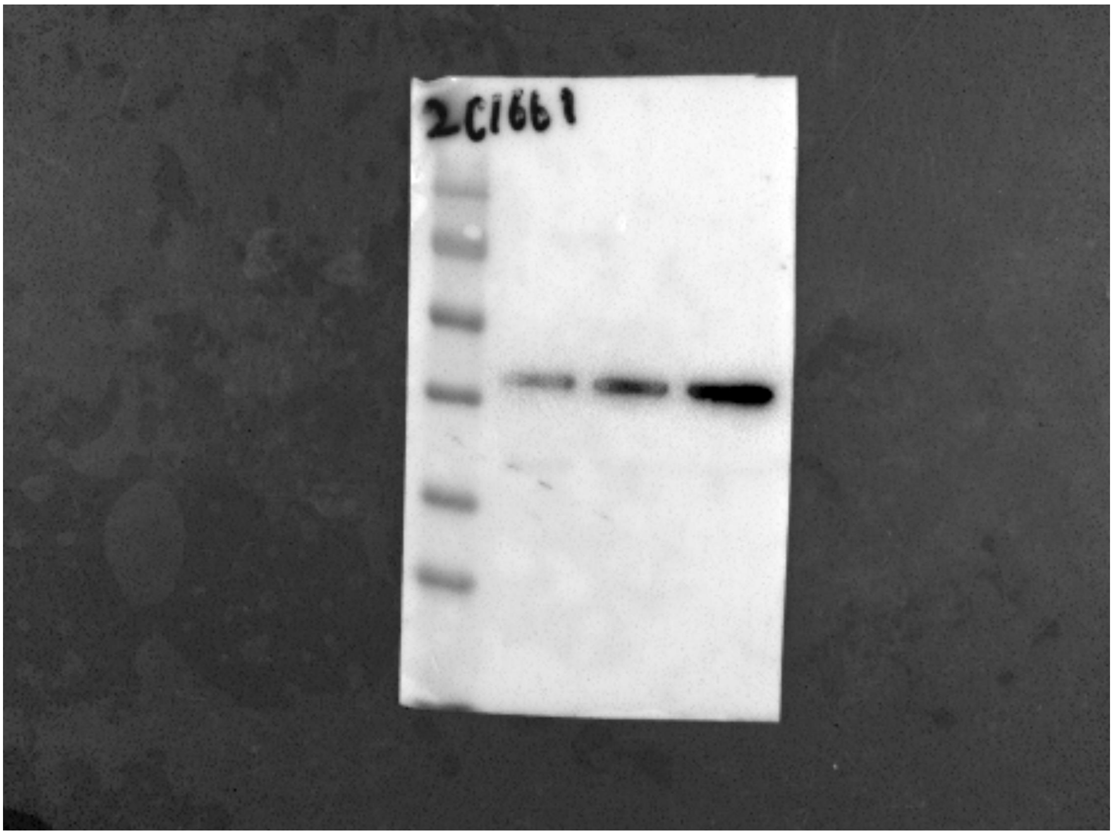

Figure legend. The western blot results of VEGF protein in C166 cells among the Control, BMSC CM, and δ-TT-BMSC CM groups.

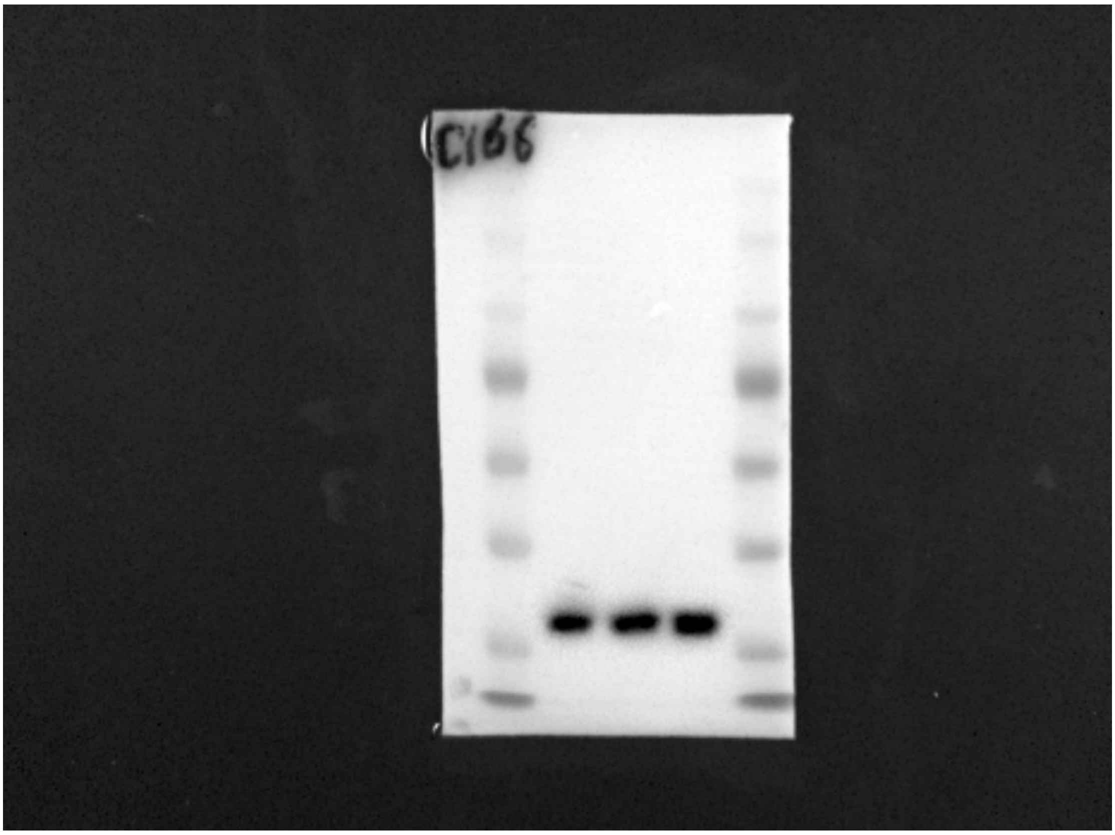

Figure legend. The western blot results of GAPDH protein in C166 cells among the Control, BMSC CM, and δ-TT-BMSC CM groups.

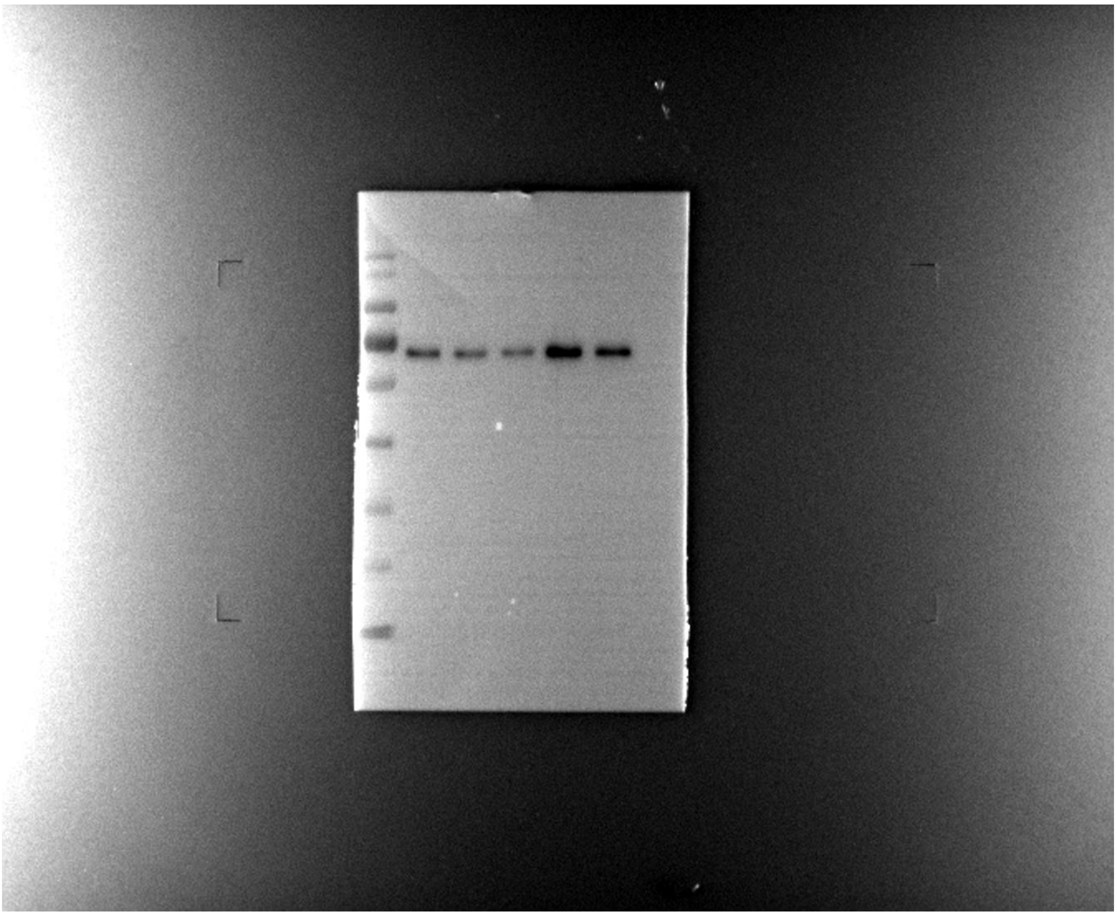

Figure legend. The western blot results of PTGS2 protein in PAM-212 cells among the Control, BMSC CM, δ-TT-BMSC CM, RSL3, and RSL3 + δ-TT-BMSC CM groups.

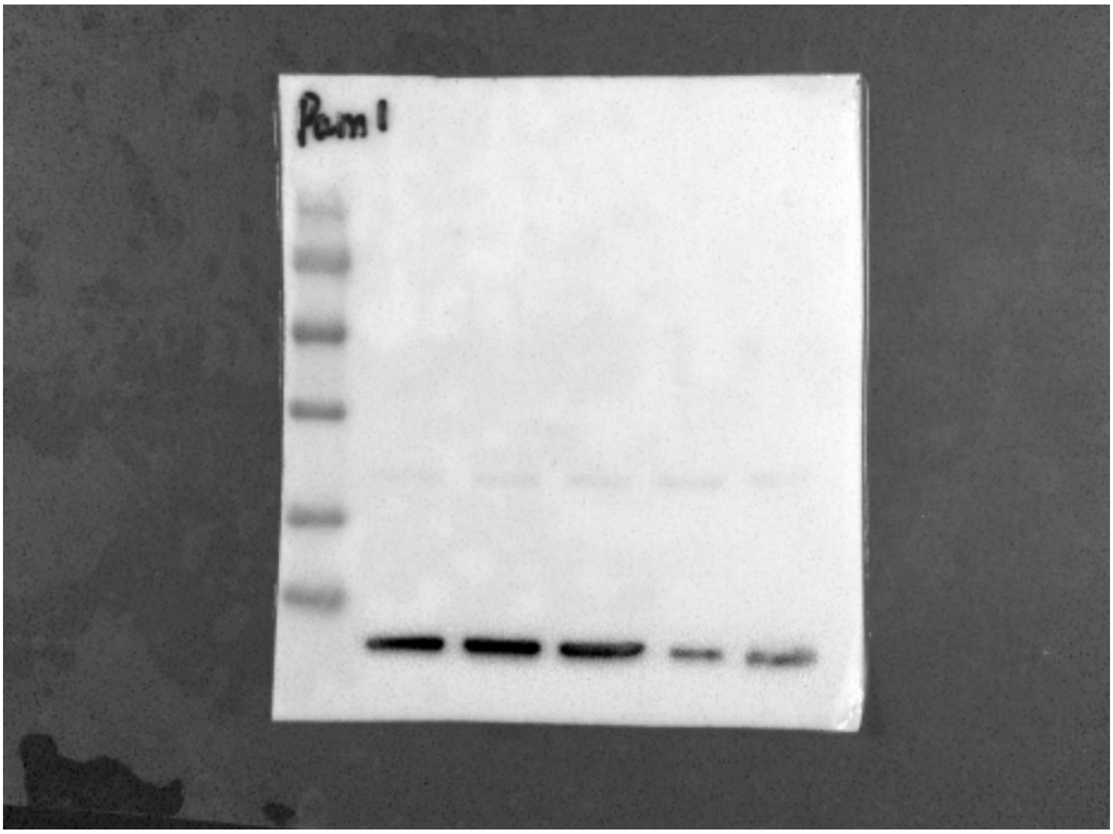

Figure legend. The western blot results of GPX4 protein in PAM-212 cells among the Control, BMSC CM, δ-TT-BMSC CM, RSL3, and RSL3 + δ-TT-BMSC CM groups.

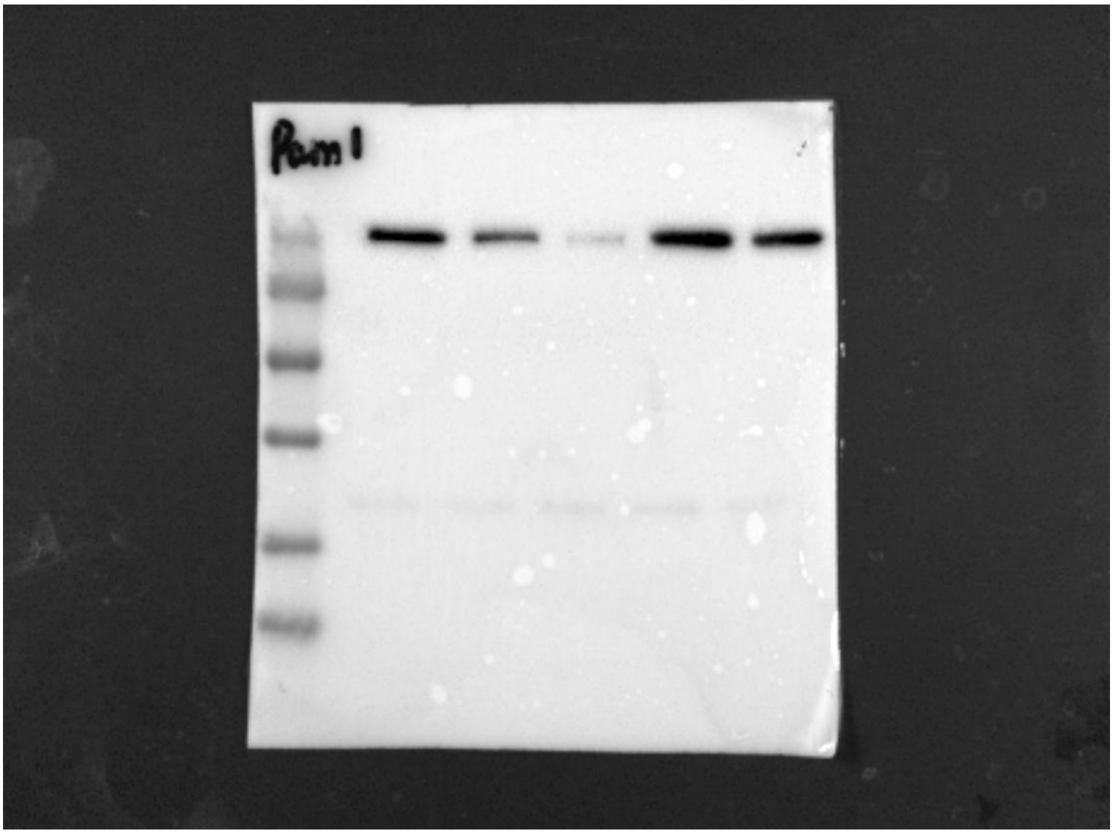

Figure legend. The western blot results of NFE2L2 protein in PAM-212 cells among the Control, BMSC CM, δ-TT-BMSC CM, RSL3, and RSL3 + δ-TT-BMSC CM groups.

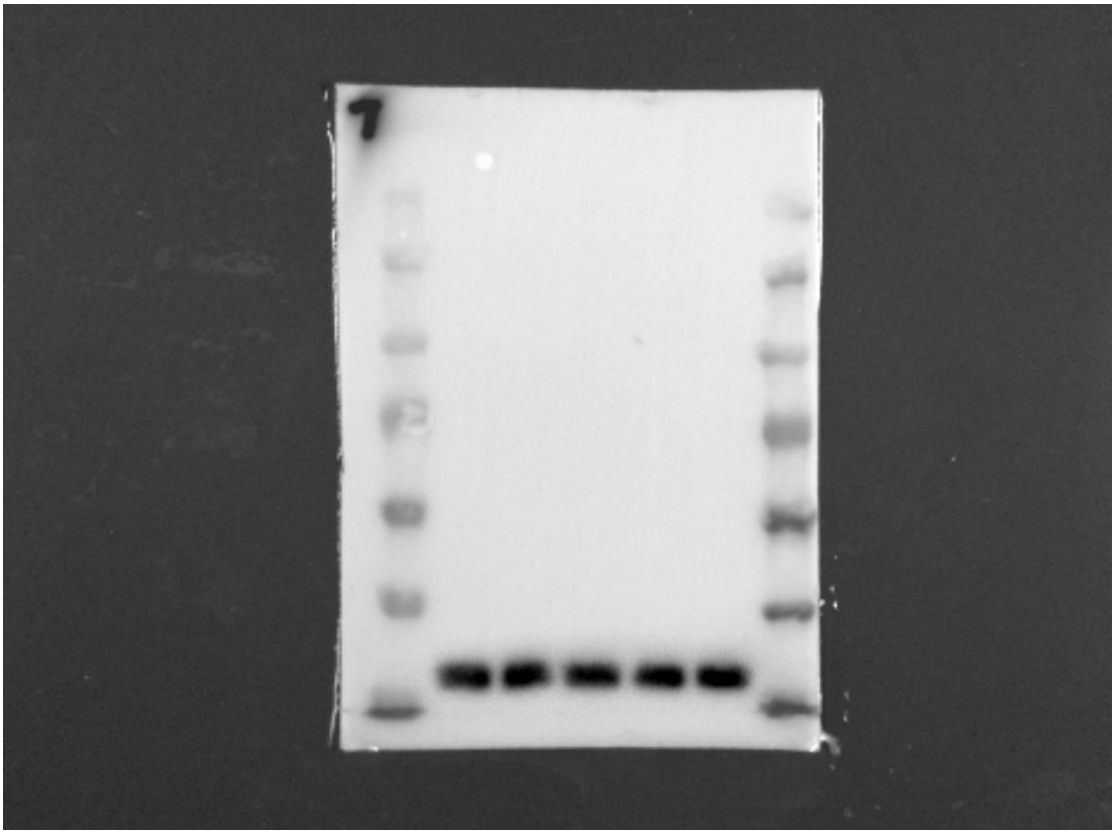

Figure legend. The western blot results of GAPDH protein in PAM-212 cells among the Control, BMSC CM, δ-TT-BMSC CM, RSL3, and RSL3 + δ-TT-BMSC CM groups.

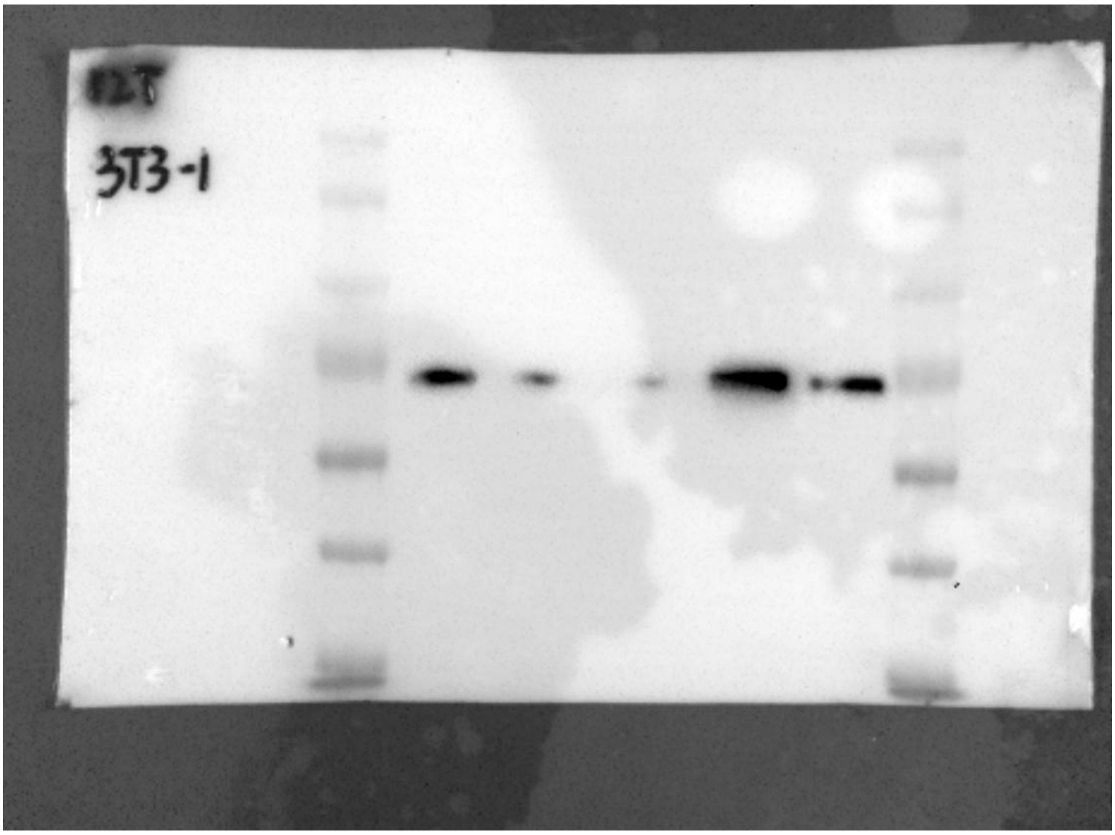

Figure legend. The western blot results of PTGS2 protein in NIH-3T3 cells among the Control, BMSC CM, δ-TT-BMSC CM, RSL3, and RSL3 + δ-TT-BMSC CM groups.

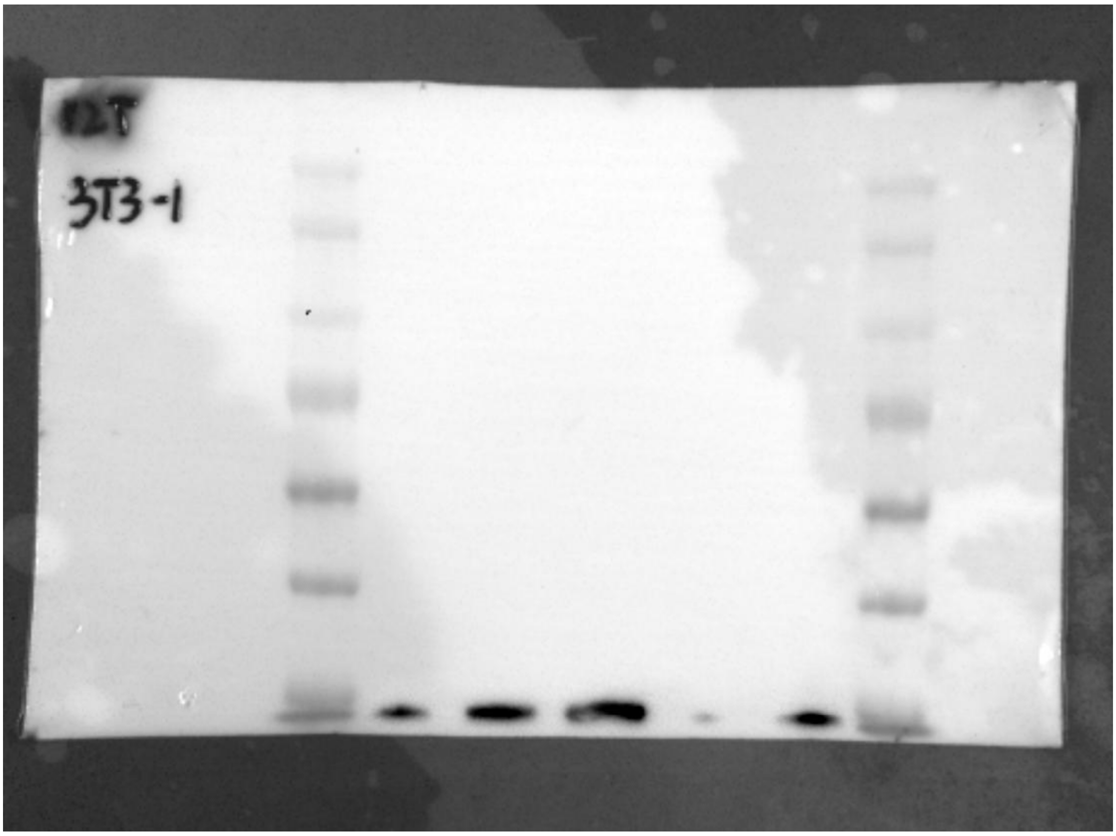

Figure legend. The western blot results of GPX4 protein in NIH-3T3 cells among the Control, BMSC CM, δ-TT-BMSC CM, RSL3, and RSL3 + δ-TT-BMSC CM groups.

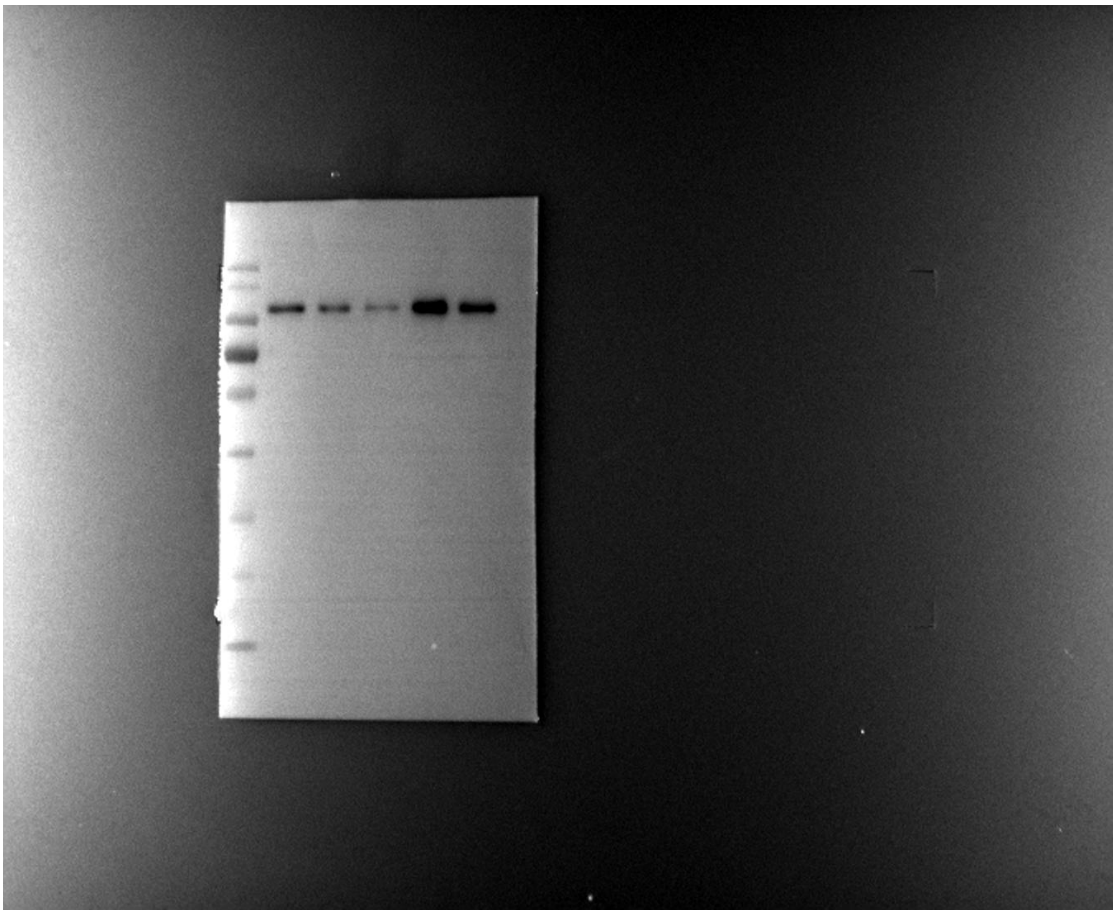

Figure legend. The western blot results of NFE2L2 protein in NIH-3T3 cells among the Control, BMSC CM, δ-TT-BMSC CM, RSL3, and RSL3 + δ-TT-BMSC CM groups.

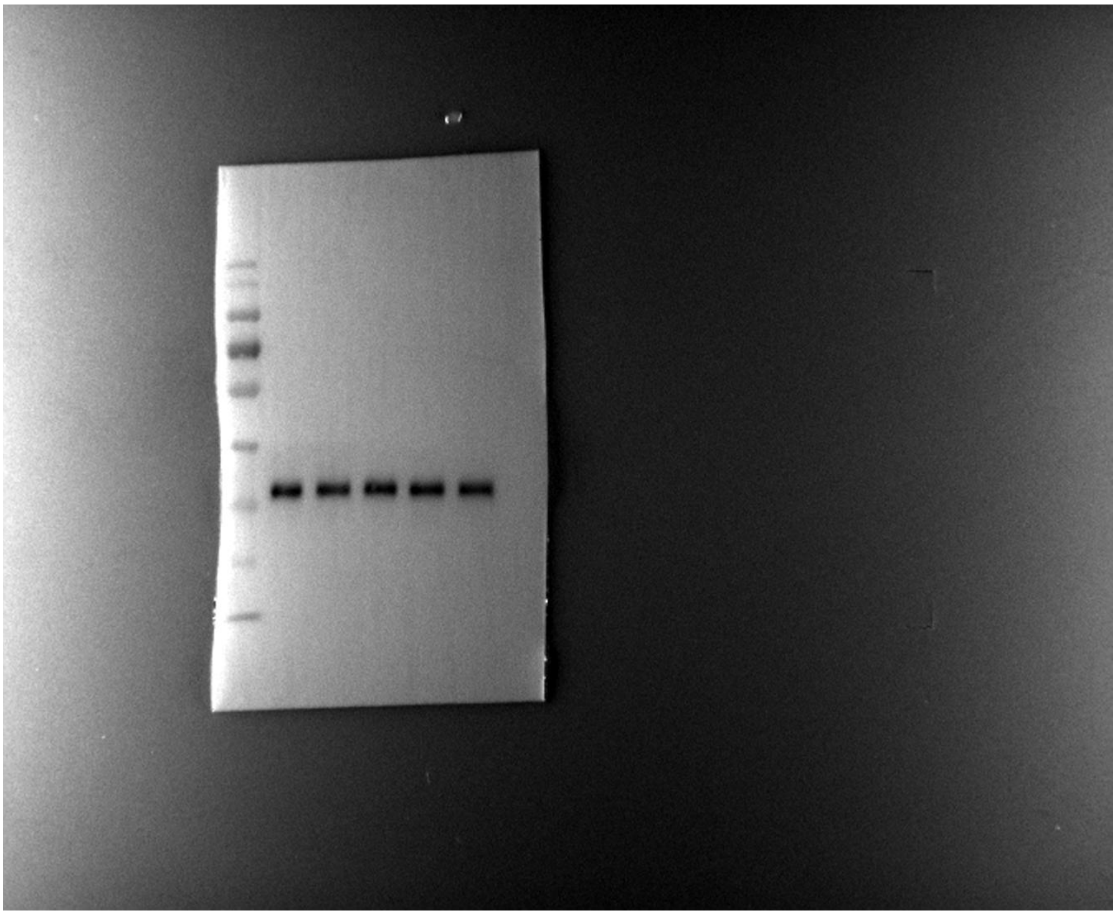

Figure legend. The western blot results of GAPDH protein in NIH-3T3 cells among the Control, BMSC CM, δ-TT-BMSC CM, RSL3, and RSL3 + δ-TT-BMSC CM groups.

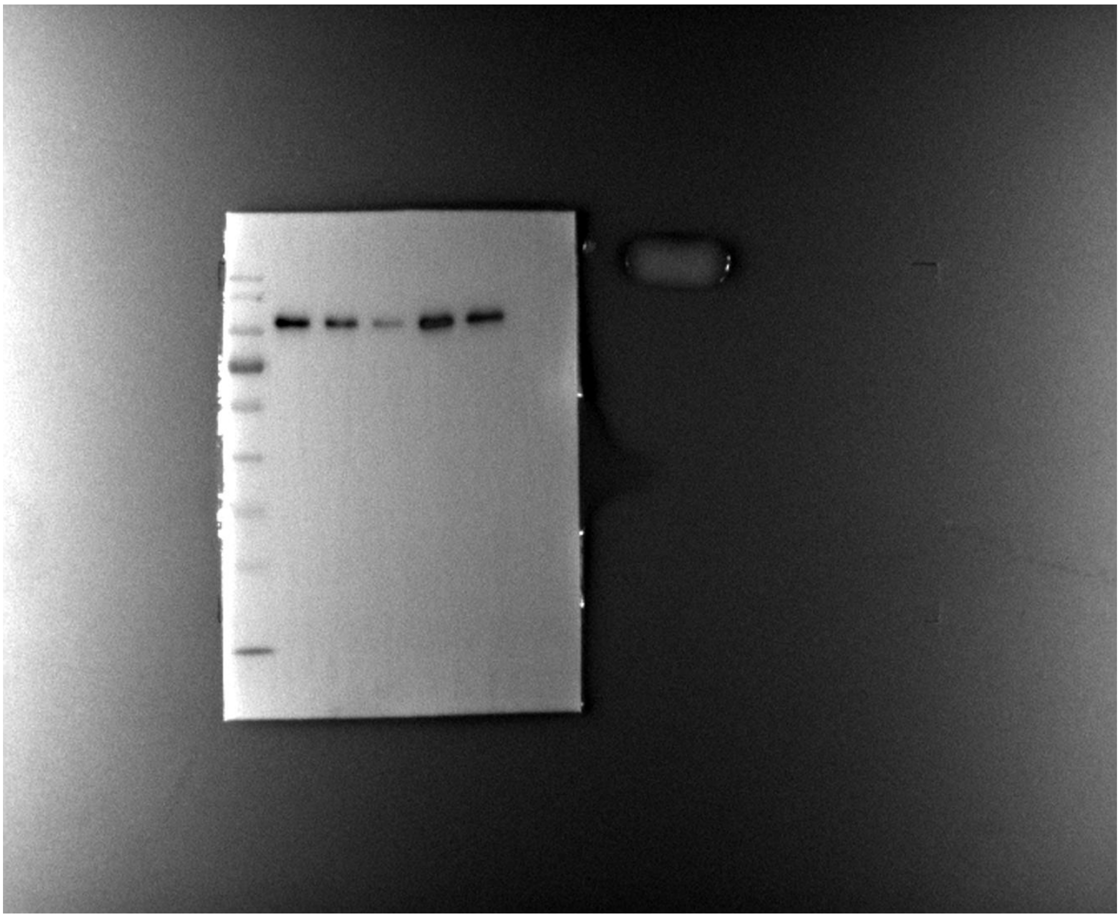

Figure legend. The western blot results of BACH1 protein in PAM-212 cells among the Control, BMSC CM, δ-TT-BMSC CM, RSL3, and RSL3 + δ-TT-BMSC CM groups.

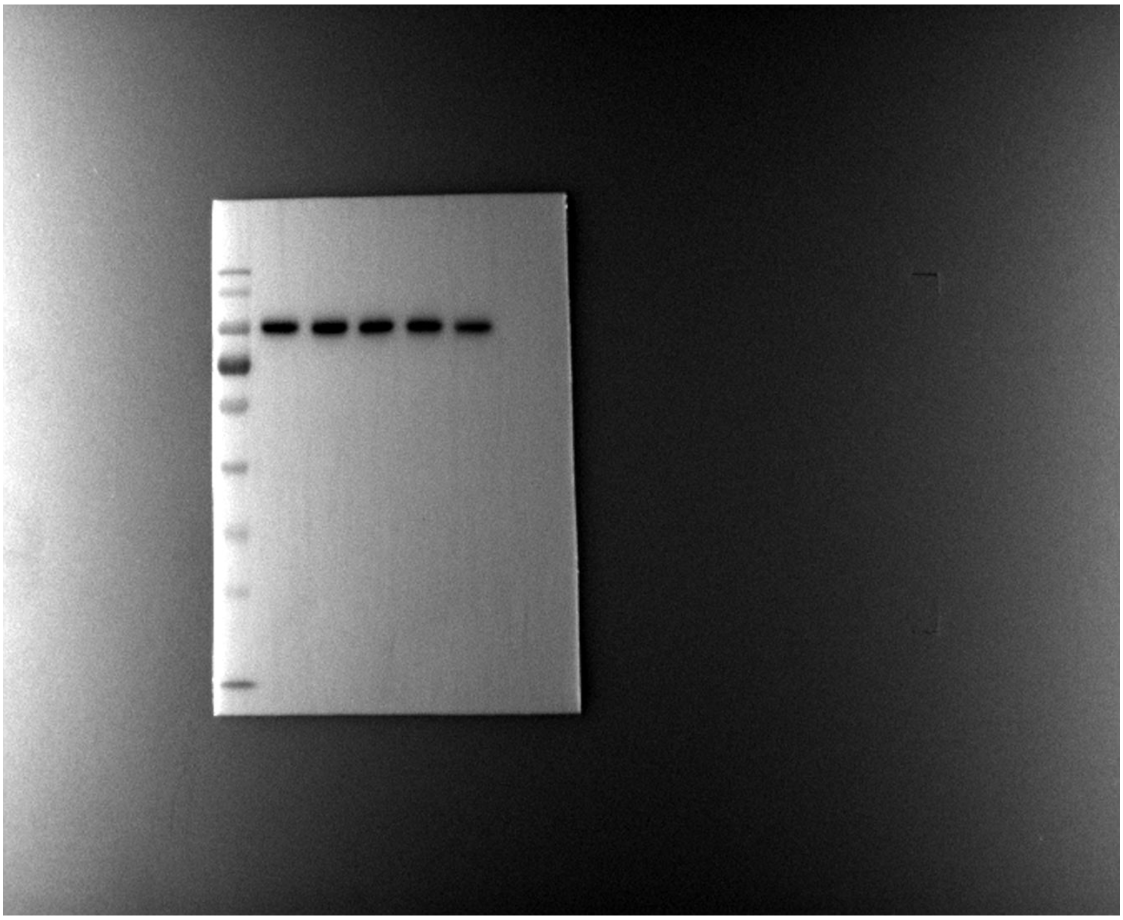

Figure legend. The western blot results of PIK3CA protein in PAM-212 cells among the Control, BMSC CM, δ-TT-BMSC CM, RSL3, and RSL3 + δ-TT-BMSC CM groups.

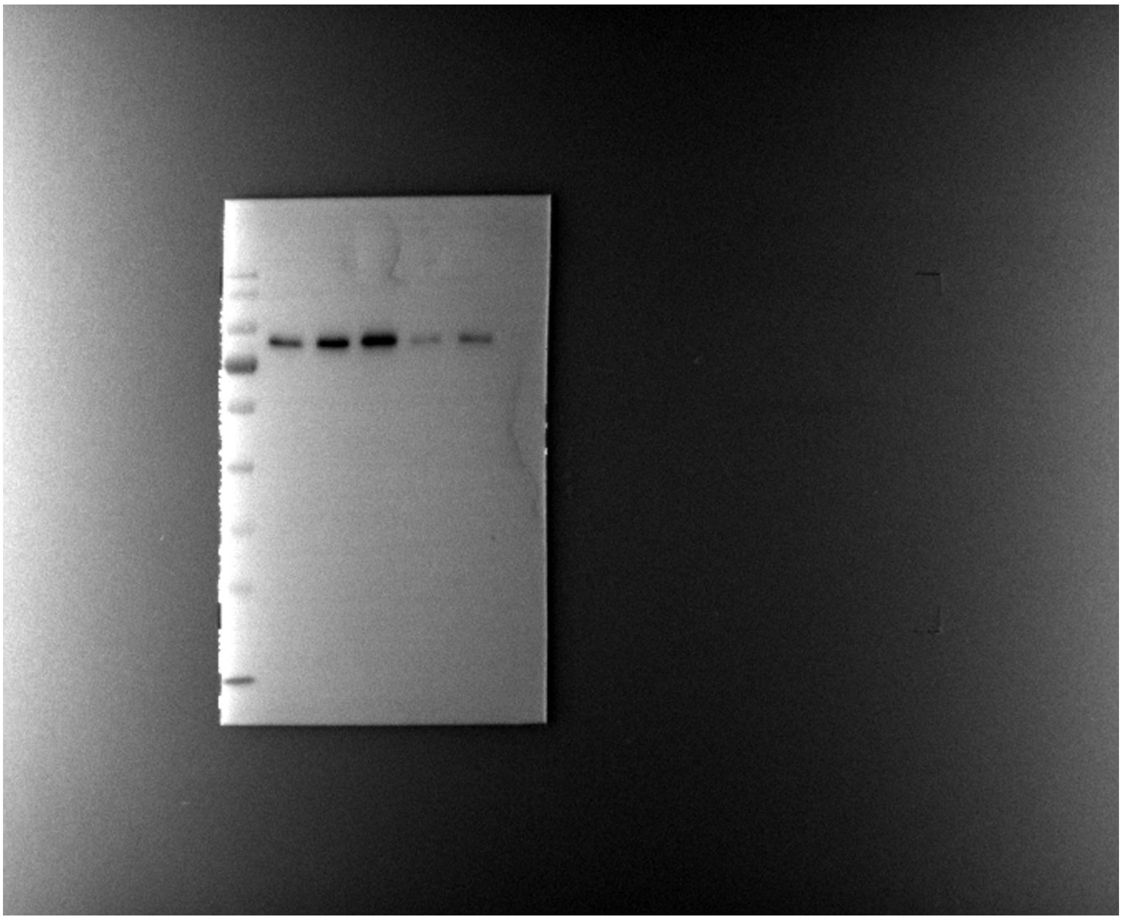

Figure legend. The western blot results of p-PI3K protein in PAM-212 cells among the Control, BMSC CM, δ-TT-BMSC CM, RSL3, and RSL3 + δ-TT-BMSC CM groups.

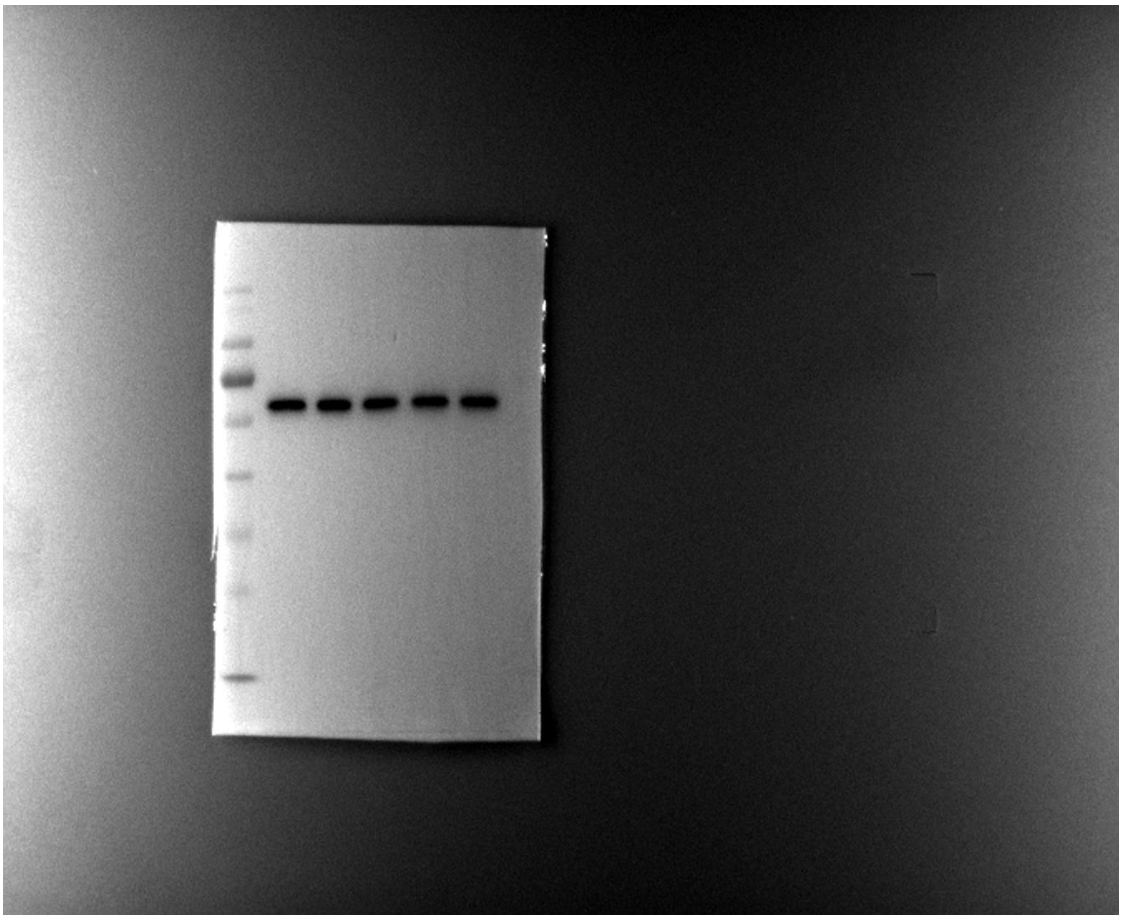

Figure legend. The western blot results of AKT protein in PAM-212 cells among the Control, BMSC CM, δ-TT-BMSC CM, RSL3, and RSL3 + δ-TT-BMSC CM groups.

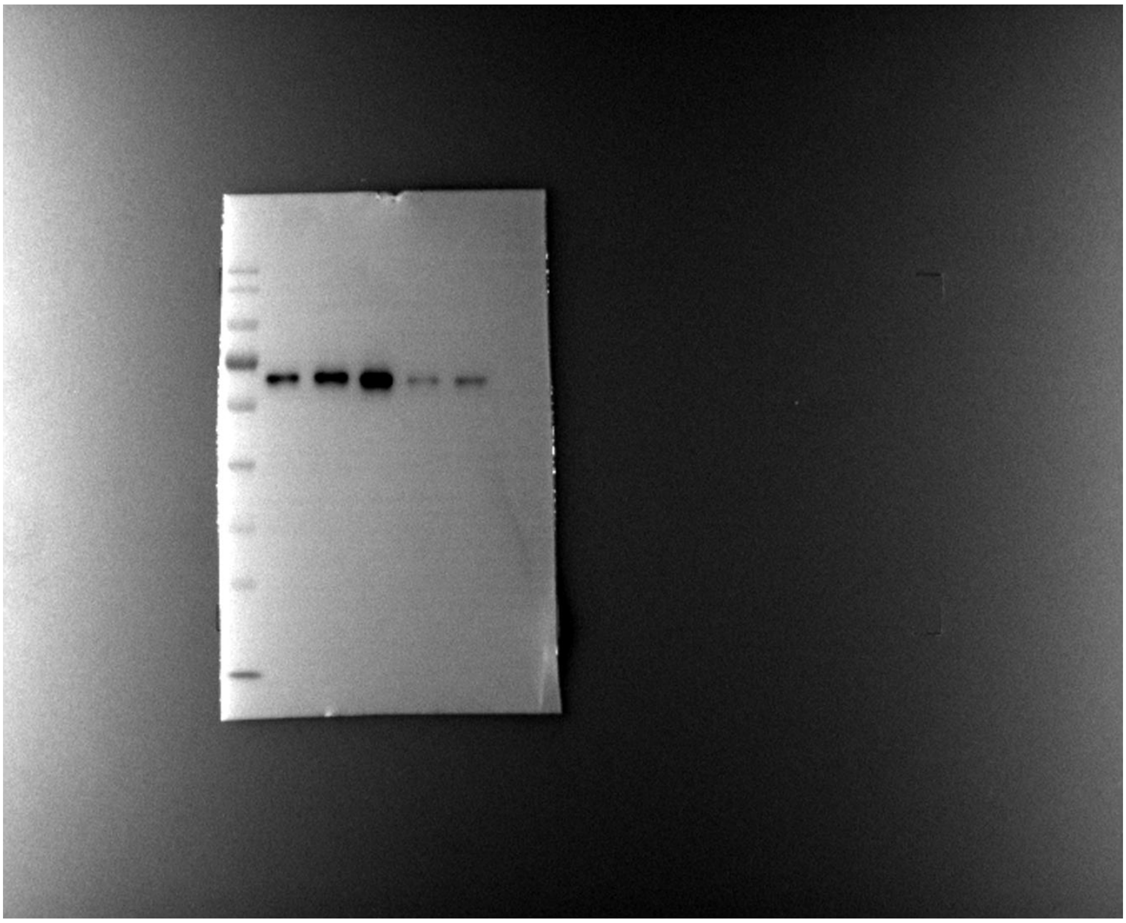

Figure legend. The western blot results of p-AKT protein in PAM-212 cells among the Control, BMSC CM, δ-TT-BMSC CM, RSL3, and RSL3 + δ-TT-BMSC CM groups.

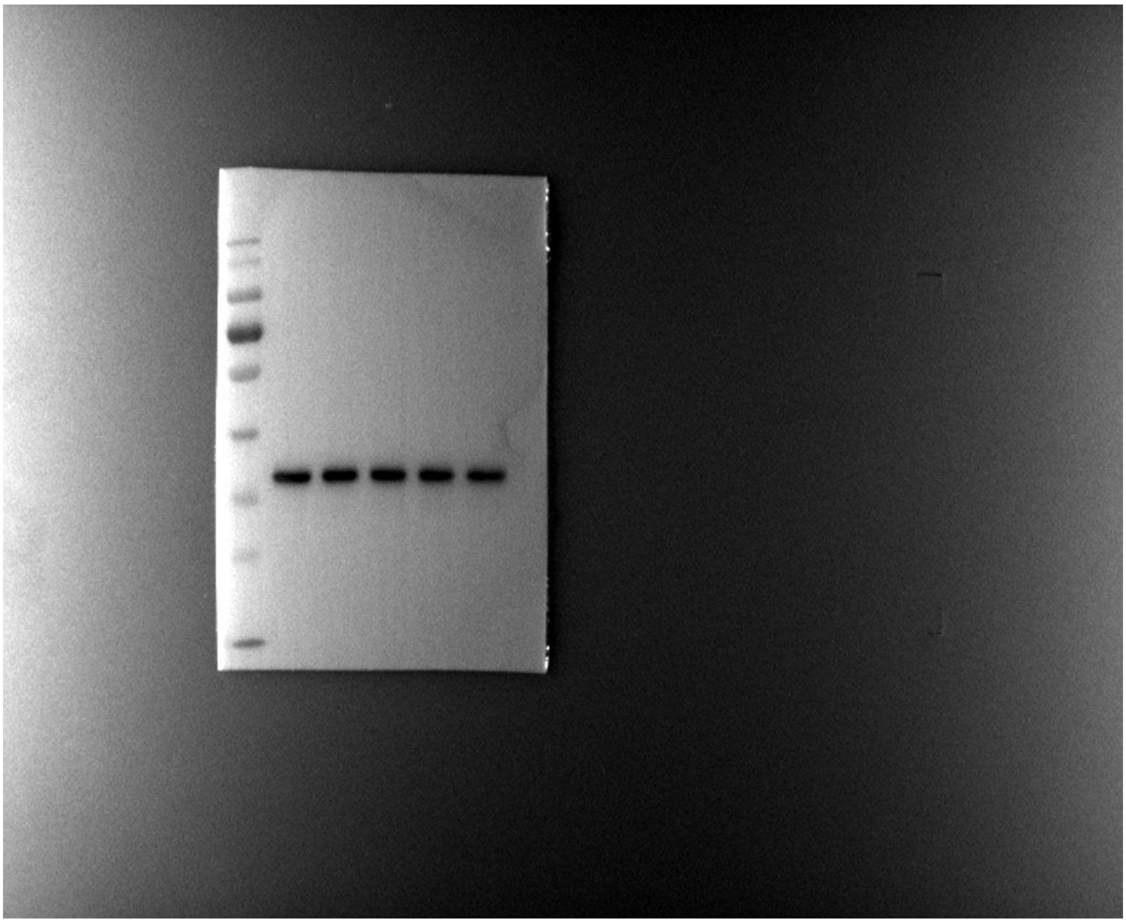

Figure legend. The western blot results of GAPDH protein in PAM-212 cells among the Control, BMSC CM, δ-TT-BMSC CM, RSL3, and RSL3 + δ-TT-BMSC CM groups.

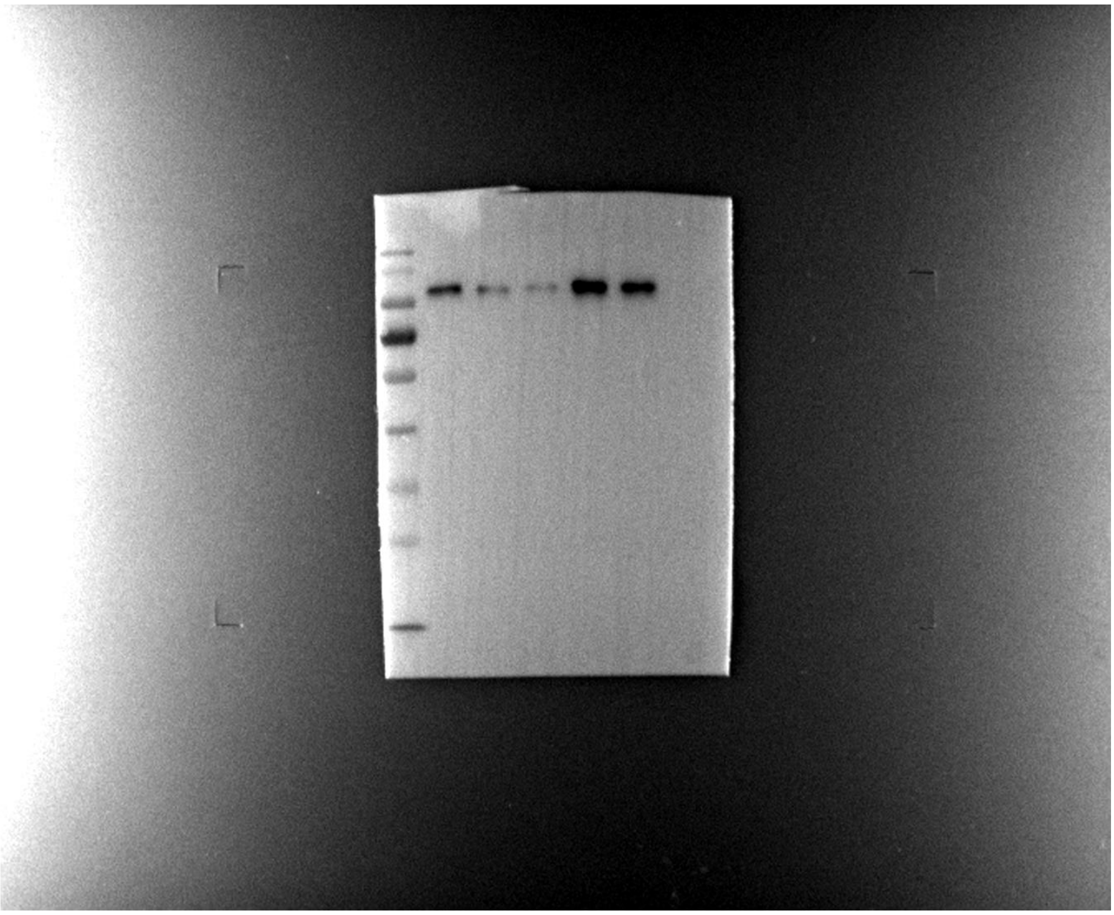

Figure legend. The western blot results of BACH1 protein in NIH-3T3 cells among the Control, BMSC CM, δ-TT-BMSC CM, RSL3, and RSL3 + δ-TT-BMSC CM groups.

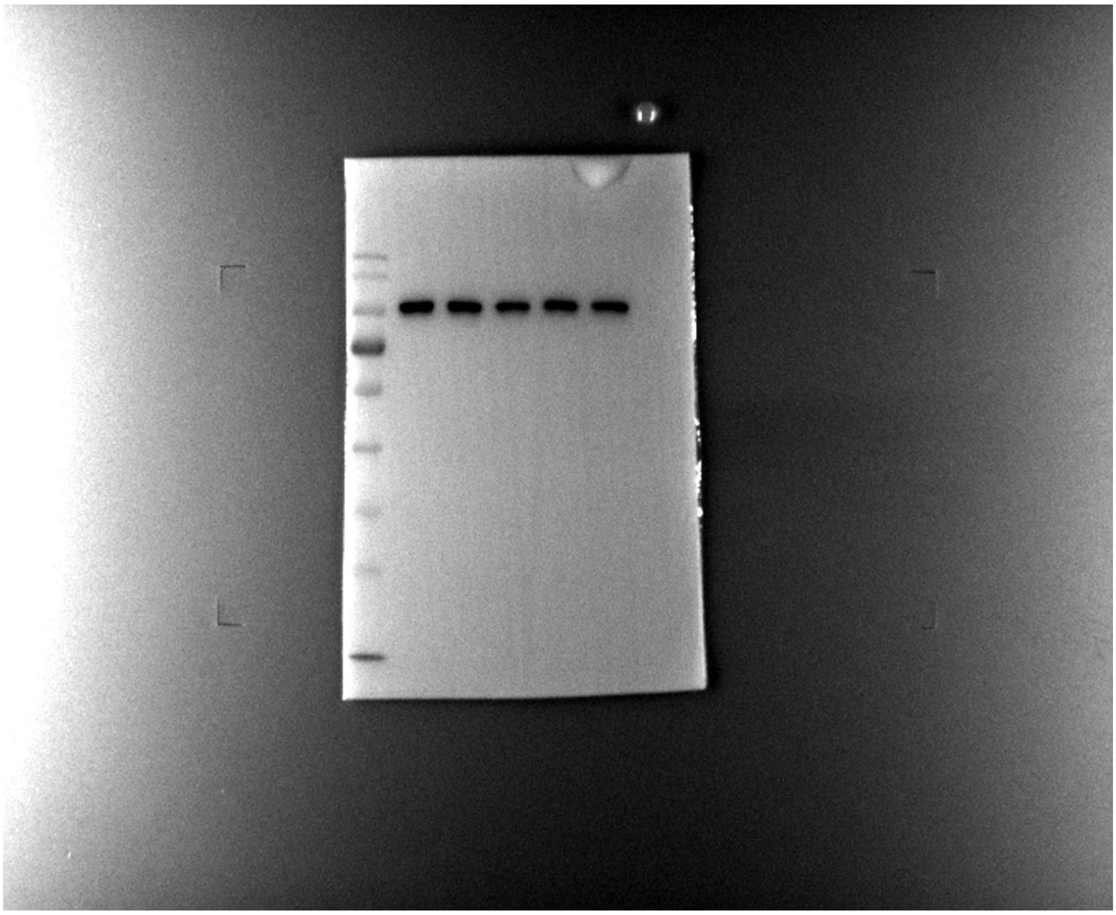

Figure legend. The western blot results of PIK3CA protein in NIH-3T3 cells among the Control, BMSC CM, δ-TT-BMSC CM, RSL3, and RSL3 + δ-TT-BMSC CM groups.

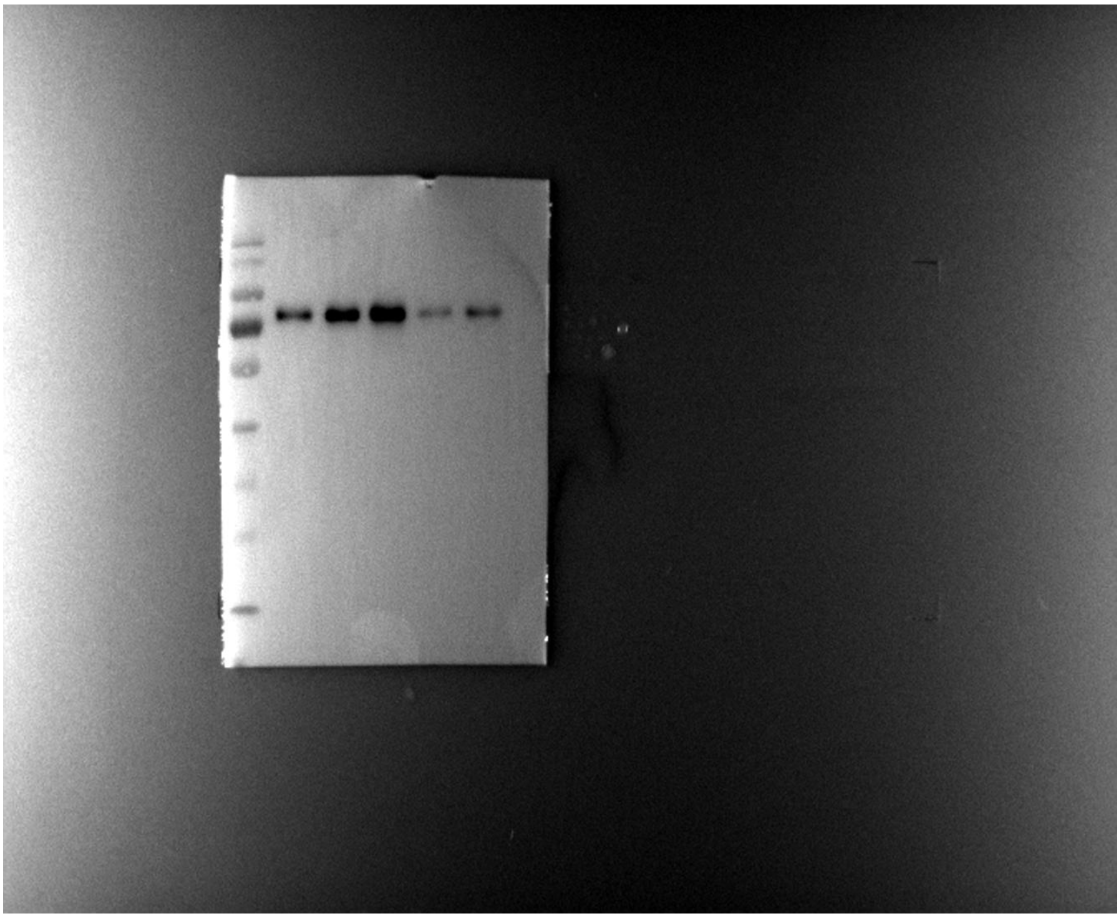

Figure legend. The western blot results of p-PI3K protein in NIH-3T3 cells among the Control, BMSC CM, δ-TT-BMSC CM, RSL3, and RSL3 + δ-TT-BMSC CM groups.

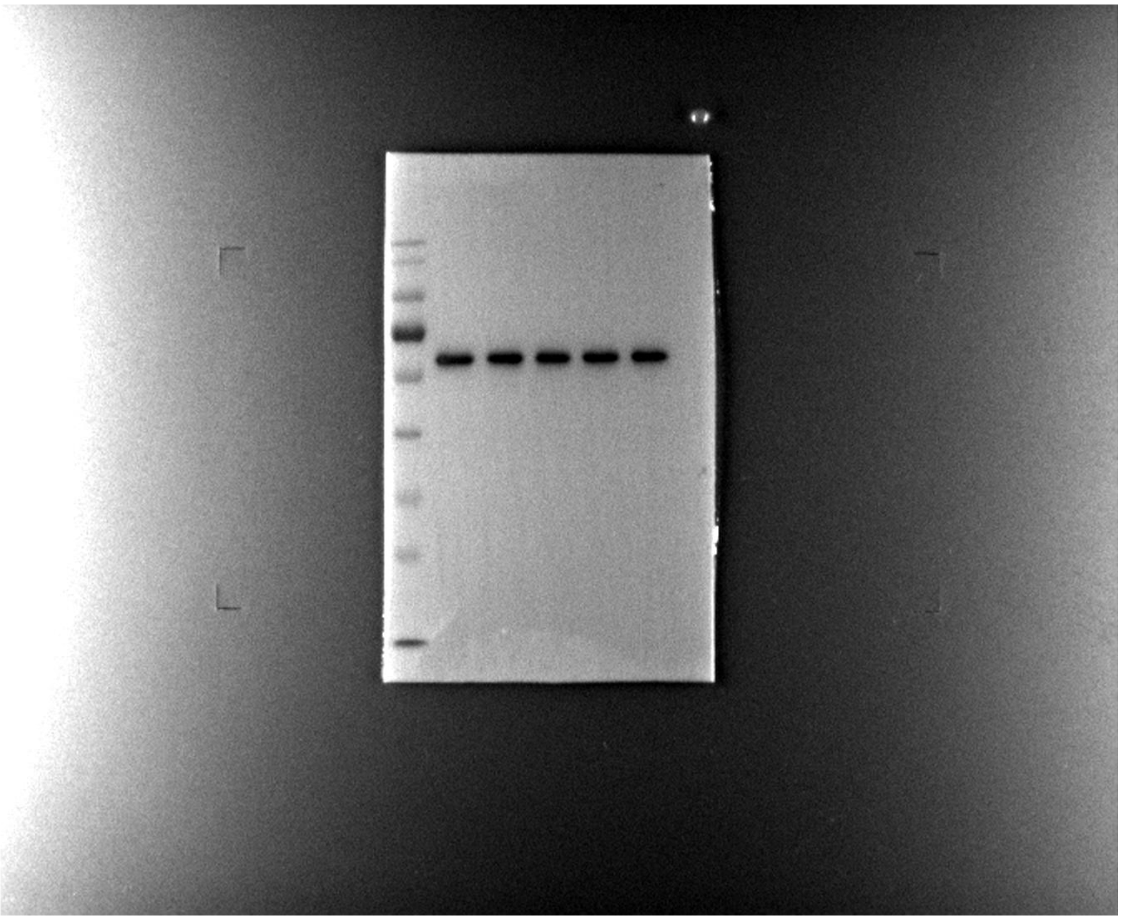

Figure legend. The western blot results of AKT protein in NIH-3T3 cells among the Control, BMSC CM, δ-TT-BMSC CM, RSL3, and RSL3 + δ-TT-BMSC CM groups.

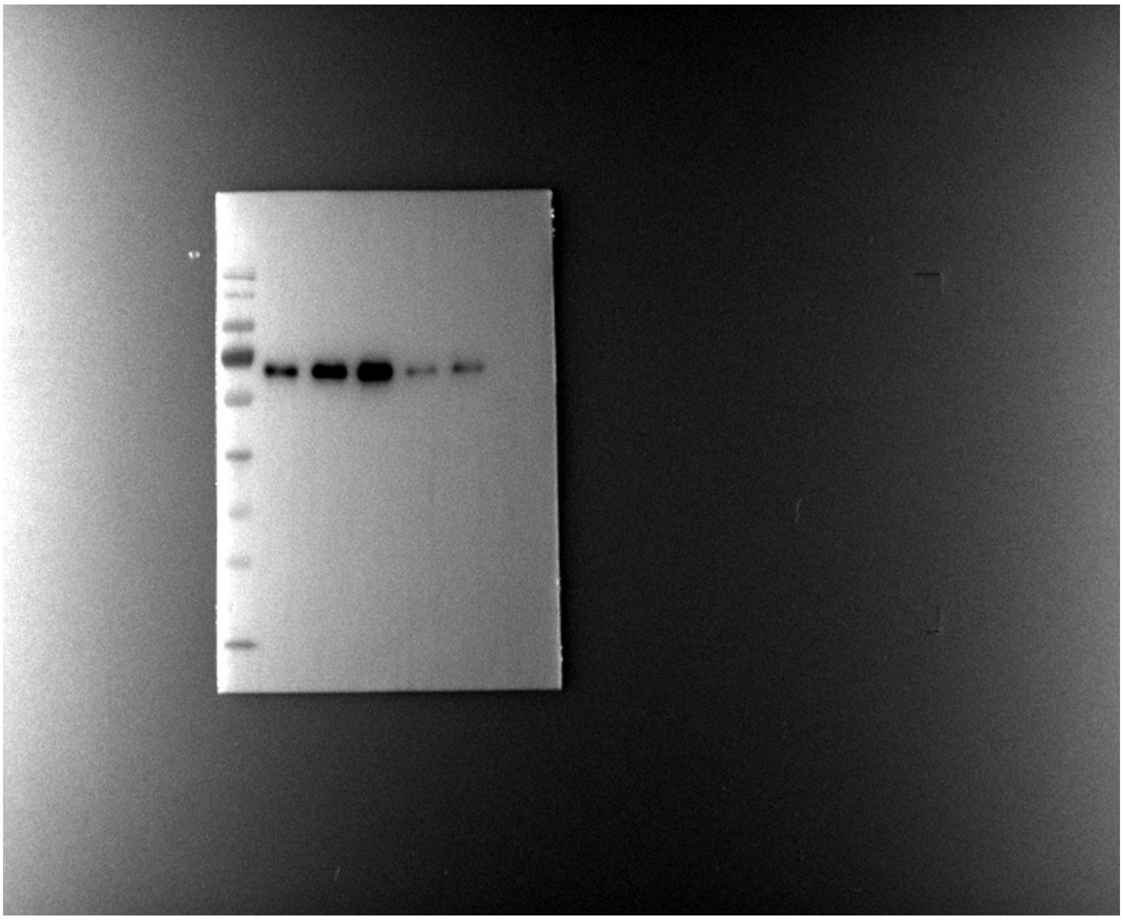

Figure legend. The western blot results of p-AKT protein in NIH-3T3 cells among the Control, BMSC CM, δ-TT-BMSC CM, RSL3, and RSL3 + δ-TT-BMSC CM groups.

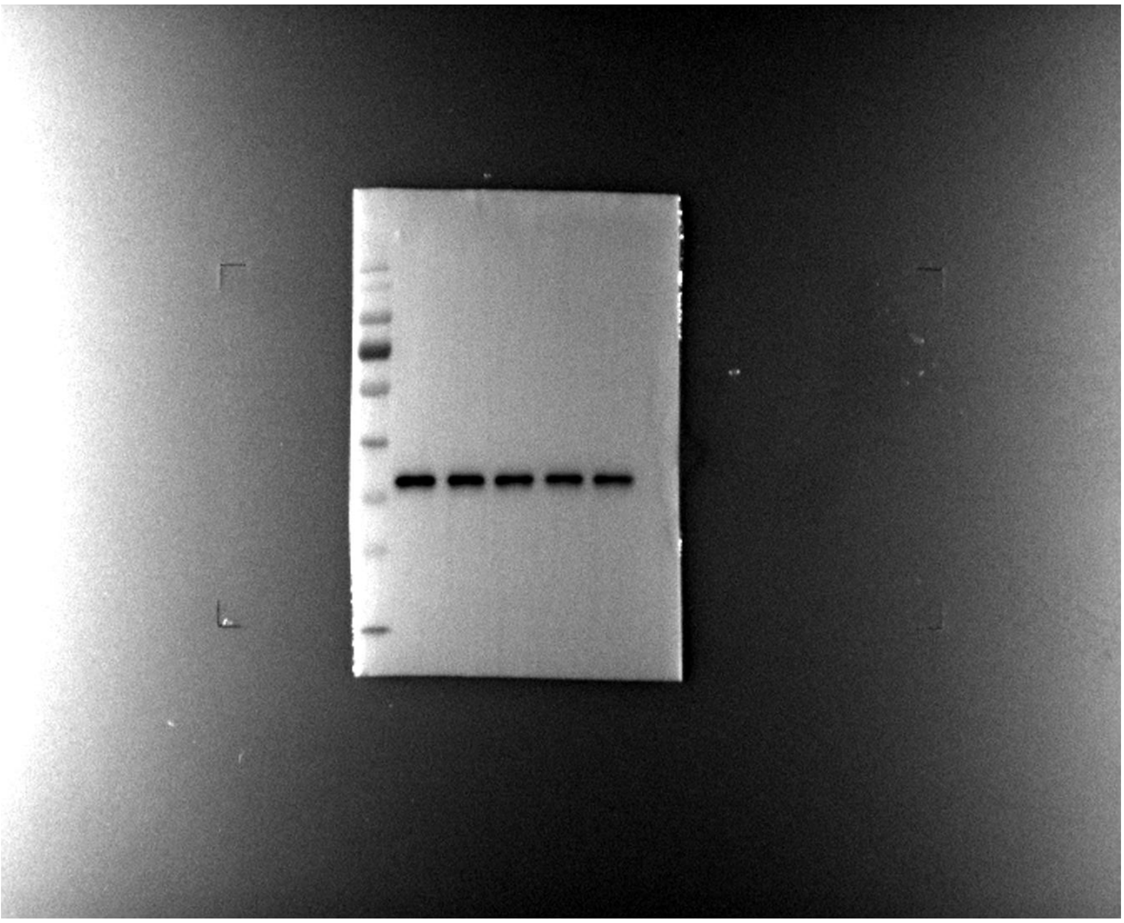

Figure legend. The western blot results of GAPDH protein in NIH-3T3 cells among the Control, BMSC CM, δ-TT-BMSC CM, RSL3, and RSL3 + δ-TT-BMSC CM groups.
