# Supplementary material for: δ-Tocotrienol preconditioning improves the capability of bone marrow-derived mesenchymal stem cells in promoting wound healing by inhibiting BACH1-related ferroptosis
Source: Cell Death Discov. 2023 Sep 22;9:349. doi: 10.1038/s41420-023-01653-1 (PMC10516898; doi:10.1038/s41420-023-01653-1)
Supplement: Supplementary file 2 — Table S1 [file 41420_2023_1653_MOESM2_ESM.docx]

**Table S1.** siRNA sequences for BACH1.

| Gene | sense 5’-3’ | antisense 5’-3’ |
| --- | --- | --- |
| si-BACH1-1 | GUAGAGCUGCAUUGUGAAAtt | UUUCACAAUGCAGCUCUACtt |
| si-BACH1-2 | CAGUCUUAGUGUUUGUUCAtt | UGAACAAACACUAAGACUGtt |
| si-BACH1-3 | CUACUGUACUUGCUUUGAAtt | UUCAAAGCAAGUACAGUAGtt |
