# Supplementary material for: δ-Tocotrienol preconditioning improves the capability of bone marrow-derived mesenchymal stem cells in promoting wound healing by inhibiting BACH1-related ferroptosis
Source: Cell Death Discov. 2023 Sep 22;9:349. doi: 10.1038/s41420-023-01653-1 (PMC10516898; doi:10.1038/s41420-023-01653-1)
Supplement: Supplementary file 3 — Table S2 [file 41420_2023_1653_MOESM3_ESM.docx]

**Table S2.** Primers for qRT-PCR.

| Gene | Forward | Reverse |
| --- | --- | --- |
| VEGF | CTGCCGTCCGATTGAGACC | CCCCTCCTTGTACCACTGTC |
| HIF-1 | ACCTTCATCGGAAACTCCAAAG | CTGTTAGGCTGGGAAAAGTTAGG |
| MMP3 | ACATGGAGACTTTGTCCCTTTTG | TTGGCTGAGTGGTAGAGTCCC |
| MMP9 | GCAGAGGCATACTTGTACCG | GCAGAGGCATACTTGTACCG |
| PDGF-B | CATCCGCTCCTTTGATGATCTT | GTGCTCGGGTCATGTTCAAGT |
| TGF-α | CACTCTGGGTACGTGGGTG | CACAGGTGATAATGAGGACAGC |
| PTGS2 | TGAGCAACTATTCCAAACCAGC | GCACGTAGTCTTCGATCACTATC |
| GPX4 | GCCTGGATAAGTACAGGGGTT | CATGCAGATCGACTAGCTGAG |
| NFE2L2 | TCTTGGAGTAAGTCGAGAAGTGT | GTTGAAACTGAGCGAAAAAGGC |
| BACH1 | TGAGTGAGAGTGCGGTATTTGC | GTCAGTCTGGCCTACGATTCT |
| GAPDH | GGAGCGAGATCCCTCCAAAAT | GGCTGTTGTCATACTTCTCATGG |
